# Supplementary material for: Global impacts of future urban expansion on terrestrial vertebrate diversity
Source: Nat Commun. 2022 Mar 25;13:1628. doi: 10.1038/s41467-022-29324-2 (PMC8956596; doi:10.1038/s41467-022-29324-2)
Supplement: Supplementary file 1 — Supplementary Information [file 41467_2022_29324_MOESM1_ESM.pdf]

Supplementary Information

Global impacts of future urban expansion on terrestrial biodiversity

Guangdong Li[1,2]*, Chuanglin Fang[1,2]*, Yingjie Li[3], Zhenbo Wang[1,2], Siao Sun[1,2], Sanwei He[4], Wei Qi[1,2], Chao Bao[1,2], Haitao Ma[1,2], Yupeng Fan[1,2], Yuxue Feng[1], Xiaoping Liu[5,6]*

Affiliations

[1] Key Laboratory of Regional Sustainable Development Modeling, Institute of Geographic Sciences and Natural Resources Research (IGSNRR), Chinese Academy of Sciences (CAS), Beijing, China

[2] College of Resources and Environment, University of Chinese Academy of Sciences, Beijing, China

[3] Center for Systems Integration and Sustainability, Department of Fisheries and Wildlife, Environmental Science and Policy Program, Michigan State University, East Lansing, MI, USA

[4] School of Public Administration, Zhongnan University of Economics and Law, Wuhan, China

[5] Guangdong Provincial Key Laboratory of Urbanization and Geo-simulation, School of Geography and Planning, Sun Yat-sen University, Guangzhou, China

[6] Southern Marine Science and Engineering Guangdong Laboratory (Zhuhai), Zhuhai, China.

* To whom correspondence should be addressed. Email: ligd@igsnrr.ac.cn (G.L.); fangcl@igsnrr.ac.cn (C.F.); liuxp3@mail.sysu.edu.cn (X.L.)

Supplementary Note 1: Research design description

Biodiversity refers to the variety of organisms from all sources, including inter alia, terrestrial, marine, and other aquatic ecosystems and the ecological complexes they comprise. Thus, biodiversity is an ecological complex that includes not only the diversity of species but also the diversity of ecosystems. Accordingly, biodiversity conservation is a multi-dimensional process that requires us not only to protect species, but also to protect their habitat and the surrounding environment. In this study, we focused on terrestrial biodiversity and combined different indicators for terrestrial biodiversity (i.e., habitat loss, habitat fragmentation, and species loss) to analyze how terrestrial biodiversity is affected by future urban expansion. We considered habitat loss and fragmentation mainly because habitat changes are closely related to species changes. Indeed, species rely on habitat to survive, and habitat loss and fragmentation are major causes of species loss. Thus, we first examined how future urban land expansion affects direct loss of natural habitat. To clarify the impacts of future urban growth on habitat loss in key and hotspot biodiversity areas, i.e., the world's most important places for species and their habitats (e.g., protected areas, biodiversity hotspots, Global 200, and the Last of the Wild areas), we also investigated the impacts of future urban expansion on biodiversity prioritization schemes. Second, we examined habitat fragmentation that is captured by edge proximity, edge density, and isolation. Finally, we focus on the species and examined the effects of future urban expansion on species richness, species abundance, and number of species. We believe that by using multiple indicators of biodiversity, we can comprehensively examine the how future urban expansion will affect biodiversity. Given the multiple dimensions of biodiversity, one indicator is not sufficient for providing a broader picture.

Supplementary Note 2: Full SSP description of urbanization pattern

The five shared socioeconomic pathways (SSPs) characterize a wide range of possible future development pathways with different trends in various domains (e.g., rate of urbanization). Below, we describe the different pathways in detail:

- **SSP1 (Sustainability):** SSP1 envisions a development path of rapid urbanization with high income growth for all country groups, including high-, middle-, and low-income countries. Urbanization is partly driven by a desire to promote environment-friendly living conditions, and compact urban form that helps improve resource efficiency [1-3]. Rural-to-urban migration is moderate. Urbanization is well managed to minimize urban sprawl and urban de-concentration [4]. Cities become stable incubators and enablers of sustainable practices [5]. Global urbanization rate is high and is expected to reach 92.6% by 2100.
- **SSP2 (Middle of the road)**: SSP2 envisions a development path of moderate urbanization and moderate income growth for all country groups[1]. Urbanization growth trends vary by region and over time, but on average they are closer to the center of expectations for future outcomes than to the upper or lower bounds of possibilities [4]. Urbanization has been particularly transformative in East and South Asia and sub-Saharan Africa. As a result of sustainable energy technologies and related designs, the transformation of cities has proceeded at different rates, with the highest rates in developed or rapidly developing cities[5]. Global urbanization rate is moderate and is expected to reach 79.7% by 2100.
- **SSP3 (Regional rivalry)**: SSP3 envisions a development path with slow urbanization for all country groups. Slow economic growth limits employment opportunities and cross-regional mobility, thus constraining the process of urbanization. Moreover, poor urban planning reduces the attractiveness of urban areas as destinations[1,6]. Developing countries face greater challenges in urbanization process, because the inequality and fragmentation in developing countries tend to cause mixed patterns of urban change (e.g., wealthier and dispersed settlements, more concentrated slum-type growth)[4]. Disadvantaged

populations, however, continue to migrate to poorly planned settlements around large urban areas, particularly in low-income countries[5]. Global urbanization rate is very low and is expected to reach 58.4% by 2100.

- **SSP4 (Inequality)**: SSP4 envisions that high-income countries will experience moderate urbanization, whereas medium- and low-income countries will experience fast urbanization. In high-income countries, moderate economic growth and appealing urban conditions in cities with a high elite population tend to support urbanization, but rapid aging due to low fertility rate has weakened rural-urban migration[7]. In contrast, high fertility rate in medium- and low-income countries produces age structures that facilitate migration from rural to urban areas. In medium-income countries, there is medium economic growth, and cities act as manufacturing centers and engines of economic growth, which facilitate rapid urbanization [8]. In low-income countries, rapid population growth, along with the shrinkage in land and other resources, has stimulated migration from rural areas to urban areas[9]. Meanwhile, large income disparities, which particularly occur between rural and urban areas, result in large flows of migration to urban areas[10]. Cities are affected by high inequality, such that the elite groups are provided with urban amenities but the rest of the population are provided with poor housing and infrastructure, which further leads to massive expansion of slums and high unemployment rates[1,11]. Spatial development patterns vary across cities, with some cities dominated by urban sprawl, whereas better planning in cities that are predominantly inhabited by the higher income classes leads to more concentrated development [4]. Urbanization rate is high and is expected to reach 91.7% by 2100.
- **SSP5 (Fossil-fueled development)**: SSP5 envisions that all country groups will experience rapid urbanization. The rapid economic growth and advancement in technologies that have enabled the development of desirable housing have made urban areas attractive destinations. Even if population growth rates decline, increases in agricultural productivity and wealth growth will lead to greater migration to cities and more urban labor force[1,6]. Unlike SSP1, however, urban

planning and land use management has difficulty to keep up with the rapid pace of urbanization in the first decades of this century, and sprawling patterns of development dominate [4]. Over time, the pace of urbanization has converged, and urban structures and forms have evolved worldwide to reflect historical patterns and prevailing local and national policies. This includes densely populated megacities in densely populated countries, as well as metropolitan areas with significant urban sprawl in other parts of the world [5]. Urbanization rate is high and is expected to reach 93.0% by 2100.

Supplementary Note 3: Global urban expansion to 2100

Based on the global projection of urban expansion with five SSPs[12], about 36~74 million hectares (Mha) of areas with urban development potential are expected to be urbanized by 2100. Moreover, the degree of future urban expansion is substantially different across five SSPs (Supplementary Fig. 1): Scenario SSP5 (fossil-fueled development pathway[13]) will undergo the greatest urban land conversion, followed by SSP2 (middle pathway between SSP1 and SSP3[14]), SSP1 (sustainable pathway[15]), and SSP4 (divided pathway[16]), whereas scenario SSP3 (regional rivalry pathway[17]) yields a minimal size of urban expansion. Notably, the United States will undergo the greatest urban land increase in scenarios SSP1, SSP2, and SSP5. However, for scenarios SSP3 and SSP4, the largest growth is predicted to occur in low-income countries located in sub-Saharan Africa (except for South Africa). Similarly, the urban growth rate (about 262 – 574% in scenarios between SSP1 and SSP4) is also the highest in these countries. In terms of spatial distribution, future urban expansion will concentrate in existing and newly-developed highly urbanized areas, such as metropolitans, and urban agglomerations around global South and North.

Supplementary Note 4: Protected Area Data

Data on the boundaries of protected areas are from the 2020 May World Database on Protected Areas[18]. We included the protected areas with specific geographic information in the database and excluded those that are only represented by points. Many of the protected areas overlap spatially but contain different IUCN categories. To eliminate these overlaps and avoid double counting of protected areas, we followed the World Database on Protected Areas User Manual (WDPA_WDOECM_Manual_1_6) and previous studies[19] to dissolve the overlapping areas into a single polygon, and designated the overlapping area as the strictest IUCN category of all protected areas at that location.

Supplementary Note 5: Global urban expansion in protected areas

Protected areas serve as the core tool and cornerstone of global biodiversity conservation, yet the protected area boundaries within the World Database on Protected Areas (WDPA) do not fully resolve inholdings (e.g., existing cities, towns, or private ownership of lands). To ensure the robustness of our results, we further utilized high-resolution (with 30m resolution) global urban expansion datasets[20-23] to identify urban expansion or human settlement changes within protected areas. Through overlapping analysis between terrestrial protected area boundaries of WDPA and urban expansion datasets (from 1972 to 2019), we still found a considerable amount of urban land or human settlement within protected areas (1,9504 km^2 and accounts for 2.04% of the total urban area of the world). Moreover, we confirm that many urban areas (or privately owned lands) are growing within the protected areas, and protected habitats within these protected areas also experienced obvious conversion. This is because 38% of the urban land use changes within protected areas were due to the conversion of natural habitats into urban land between 1992 and 2015 based on the CCI-LC data. Therefore, there is an urgent need to gradually reduce human disturbance and urban expansion within the protected areas, and to explore the coordinated symbiosis of urban development and biodiversity conservation.

Supplementary Note 6: Biodiversity Hotspots Data

Biodiversity hotspots are places on Earth that are both rich in biological resources and deeply threatened. To meet the criteria of a biodiversity hotspot, an area must (a) include at least 1,500 endemic vascular plant species that exist nowhere else on the planet, and (b) have 30% or less of its original natural vegetation (i.e., threatened)[24]. Based on these two criteria, 36 ecoregions have been identified as biodiversity hotspots[25], and the success of species conservation in these ecoregions has greatly impact in protecting global biodiversity.

Supplementary Note 7: Global 200 Data

WWF's Global 200 project[26] examined global patterns of biodiversity to identify the Earth's terrestrial, freshwater, and marine ecoregions that possess exceptional biodiversity and represent their ecosystems. This project placed each ecoregion on Earth in a system of 30 biomes and biogeographic realms to facilitate further representation. It also compared the biodiversity characteristics (e.g., species richness, endemic species, unusual higher taxa, unusual ecological or evolutionary phenomena, and global rarity of habitats) of different ecoregions to evaluate their irreplaceability. This process yielded 238 ecoregions (i.e., the Global 200), including 142 terrestrial, 53 freshwater and 43 marine priority ecoregions (https://www.worldwildlife.org/publications/global-200). Effectively protecting these ecoregions will help preserve the planet's most representative biodiversity habitats.

Supplementary Note 8: Last of the Wild Areas Data

According to the Human Footprint Index dataset, Last of the Wild (version 2)[27] represents the areas of major terrestrial biomes that are least affected by humans or wild areas. The wildest areas in each biome were defined as areas with a Human Footprint Index value of less than or equal to 10. The 10 largest polygons with more than 5 square kilometers within each biome were selected and identified as wild areas. Last of the Wild provides up-to-date geographical projection maps of wild areas that can be used for designing wildlife conservation programs, effective management of natural resources, and research on the relationship between humans and their environment.

Supplementary Note 9: PREDICTS

The modelled mean estimates of the relative percentage of biodiversity change for each Land System were based on biodiversity data from the databased of the PREDICT (Projecting Responses of Ecological Diversity In Changing Terrestrial Systems) Project [28]. The PREDICTS collated inter-site comparisons of ecological assemblage composition from published studies or unpublished datasets to study the impact of human activity on more than one named taxon. Extracts from this database were completed on April 28, 2015. This extract includes 2.38 million records from 413 published sources or unpublished datasets, documenting the occurrence or abundance of 39123 species at 18659 sites in all 14 terrestrial biomes in the world. The site-level data used to build the models are publicly available from the Natural History Museum's Data Portal (https://www.nhm.ac.uk/our-science/our-work/biodiversity/predicts.html). These data are reasonably representative of the major taxa and terrestrial biomes. For studies where sampling effort varied across sampling sites, abundance values were corrected through dividing them by sampling effort (i.e., assuming that abundance increases linearly with effort). The database includes more than 1% of the total number of all described species, and more than 1% of described species within many taxa. Hudson et al.[18] only present data in which (a) species abundance, species occurrence, or species richness was measured at two or more sampling sites and/or times, and (b) all sites were sampled using the same procedure and the same workload or site-specific workload data. They preferentially use the geographic coordinates in the file or those provided by the data provider; however, in cases where the coordinates are not available, they geo-reference from the map in the file. The final dataset was drawn from 378 studies and two unpublished datasets. The resulting dataset contains data on 26,953 species at 11,525 sites. A full description of how datasets are assembled and managed was provided elsewhere[28].

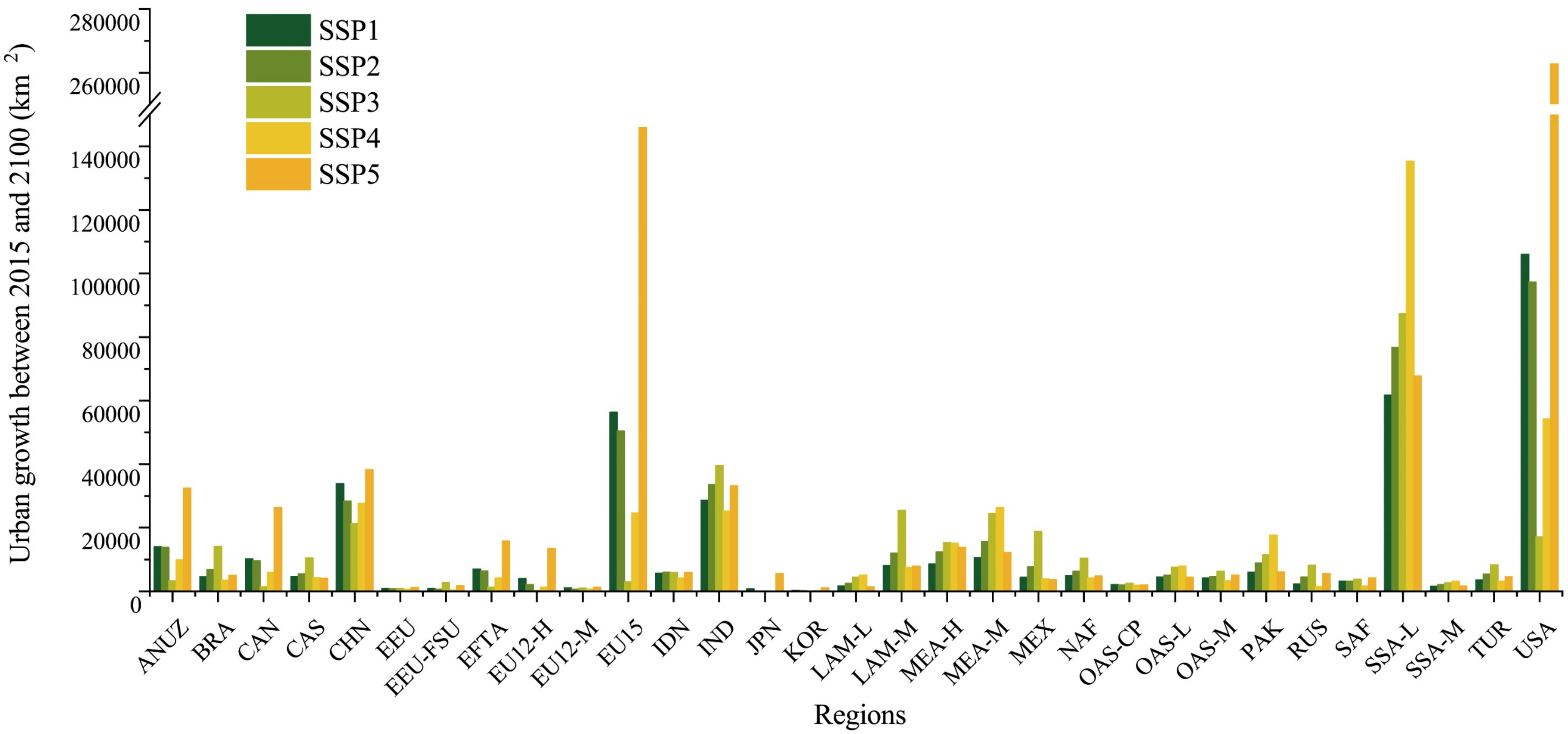

Supplementary Fig. 1 Urban expansion projections by 2100 under SSP scenarios. These 32 macro regions are defined as follows: ANUZ = Australia and New Zealand. BRA = Brazil. CAN = Canada. CAS = countries in Central Asia, including Armenia, Azerbaijan, Georgia, Kazakhstan, Kyrgyzstan, Tajikistan, Turkmenistan, and Uzbekistan. CHN = China (Mainland, Hongkong, Macao; excl. Taiwan), including China, Hong Kong SAR (China), Macao SAR (China).

EEU = Eastern Europe (excl. former Soviet Union and EU member states), including Albania, Bosnia and Herzegovina, Croatia, Montenegro, Serbia, The former Yugoslav Republic of Macedonia. EEU-FSU = Eastern Europe, former Soviet Union (excl. Russia and EU members). Belarus, Republic of Moldova, Ukraine. EFTA = Iceland, Norway, and Switzerland. EU12-H = New EU member states that joined as of 2004 - high income. Cyprus, Czech Republic, Estonia, Hungary, Malta, Poland, Slovakia, Slovenia. EU12-M = medium-income New EU member states that joined as of 2004, including Bulgaria, Latvia, Lithuania, and Romania. EU15 = European Union member states that joined prior to 2004, including Austria, Belgium, Denmark, Finland, France, Germany, Greece, Ireland, Italy, Luxembourg, Netherlands, Portugal, Spain, Sweden, and United Kingdom. IDN = Indonesia. IND = India. JPN = Japan. KOR = Republic of Korea. LAM-L = low-income countries in Latin America (excl. Brazil, Mexico), including Belize, Guatemala, Haiti, Honduras, and Nicaragua. LAM-M = medium- and high-income countries in Latin America (excl. Brazil, Mexico), including Antigua and Barbuda, Argentina, Bahamas, Barbados, Bermuda, Bolivia (Plurinational State of), Chile, Colombia, Costa Rica, Cuba, Dominica, Dominican Republic, Ecuador, El Salvador, French Guiana, Grenada, Guadeloupe, Guyana, Jamaica, Martinique, Netherlands Antilles, Panama, Paraguay, Peru, Saint Kitts and Nevis, Saint Lucia, Saint Vincent and the Grenadines, Suriname, Trinidad and Tobago, Uruguay, and Venezuela (Bolivarian Republic of). MEA-H = high-income countries in Middle East Asia, including Bahrain, Israel, Kuwait, Oman, Qatar, Saudi Arabia, and United Arab Emirates. MEA-M = low- and medium-income countries in Middle East Asia, including Iran (Islamic Republic of), Iraq, Jordan, Lebanon, Occupied Palestinian Territory, Syrian Arab Republic, and Yemen. MEX = Mexico. NAF = countries in North Africa, including Algeria, Egypt, Libyan Arab Jamahiriya, Morocco, Tunisia, and Western Sahara. OAS-CPA = countries in Other Asia (i.e., former Centrally Planned Asia), including Cambodia, Lao People's Democratic Republic, Mongolia, and Viet Nam. OAS-L = low-income countries in Other Asia, including Bangladesh, Democratic People's Republic of Korea, Fiji, Micronesia (Fed. States of), Myanmar, Nepal, Papua New Guinea, Philippines, Samoa, Solomon Islands, Timor-Leste, Tonga, and Vanuatu. OAS-M = medium- and high-income countries in Other Asia, including Bhutan, Brunei Darussalam, French Polynesia, Guam, Malaysia, Maldives, New Caledonia, Singapore, Sri Lanka, and Thailand. PAK = Pakistan and Afghanistan. RUS = Russian Federation. SAF = South Africa. SSA-L = low-income countries in Subsahara Africa (excl. South Africa), including Benin, Burkina Faso, Burundi, Cameroon, Cape Verde, Central African Republic, Chad, Comoros, Congo, Côte d`Ivoire, Democratic Republic of the Congo, Djibouti, Eritrea, Ethiopia, Gambia, Ghana, Guinea, Guinea-Bissau, Kenya, Lesotho, Liberia, Madagascar, Malawi, Mali, Mauritania, Mozambique, Niger, Nigeria, Rwanda, Sao Tome and Principe, Senegal, Sierra Leone, Somalia, South Sudan, Sudan, Swaziland, Togo, Uganda, United Republic of Tanzania, Zambia, and Zimbabwe. SSA-M = medium- and high-income countries of Subsahara Africa (excl. South Africa), including Angola, Botswana, Equatorial Guinea, Gabon, Mauritius, Mayotte, Namibia, Réunion, and Seychelles. TUR = Turkey. TWN = Taiwan. USA = United States of America. Includes: Puerto Rico, United States Virgin Islands, United States of America.

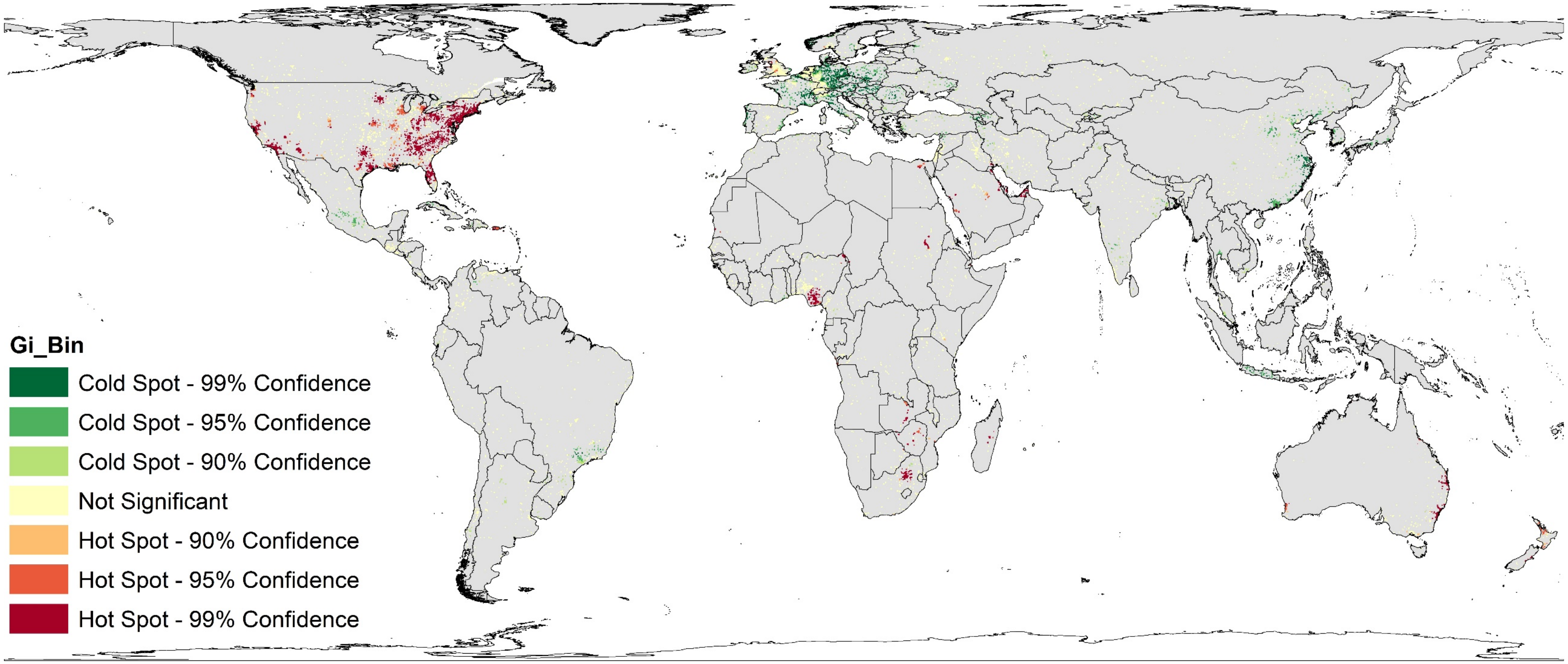

Supplementary Fig. 2 Future hot spots and cold spots of habitat loss due to urban expansion under SSP1. The Hot Spot Analysis (Getis-Ord Gi*) tool in ArcGIS Pro 2.5 was used. This tool identifies statistically significant spatial clusters of high values (hot spots) and low values (cold spots). Potential habitat loss due to future urban expansion was input variable. The Gi_Bin identifies statistically significant hot and cold spots. Statistical significance was based on the *P*-value and *Z*-score (two-sided), and no adjustments were made for multiple comparisons.

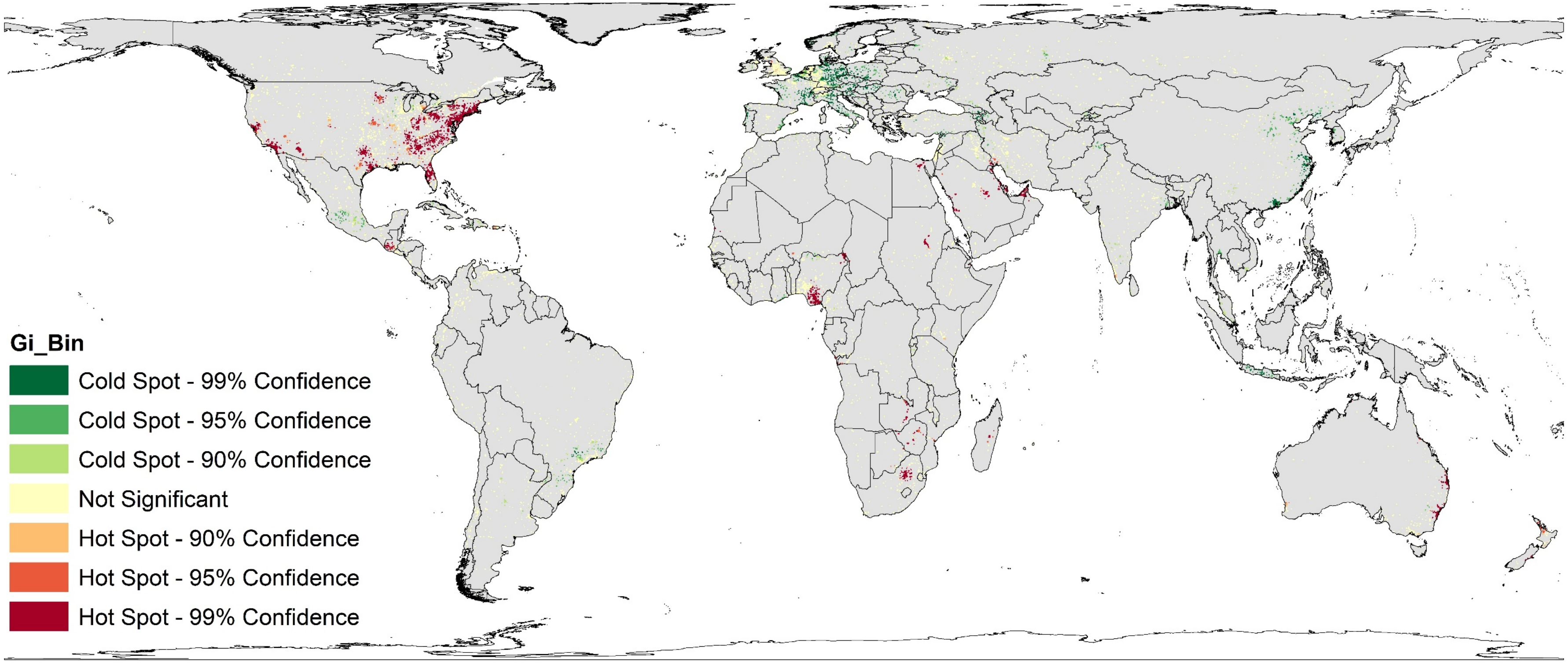

Supplementary Fig. 3 Future hot-spots and coldspots of habitat loss due to urban expansion under SSP2. The Hot Spot Analysis (Getis-Ord Gi*) tool in ArcGIS Pro 2.5 was used. This tool identifies statistically significant spatial clusters of high values (hot spots) and low values (cold spots). Potential habitat loss due to future urban expansion was input variable. The Gi_Bin identifies statistically significant hot and cold spots. Statistical significance was based on the *P*-value and *Z*-score (two-sided), and no adjustments were made for multiple comparisons.

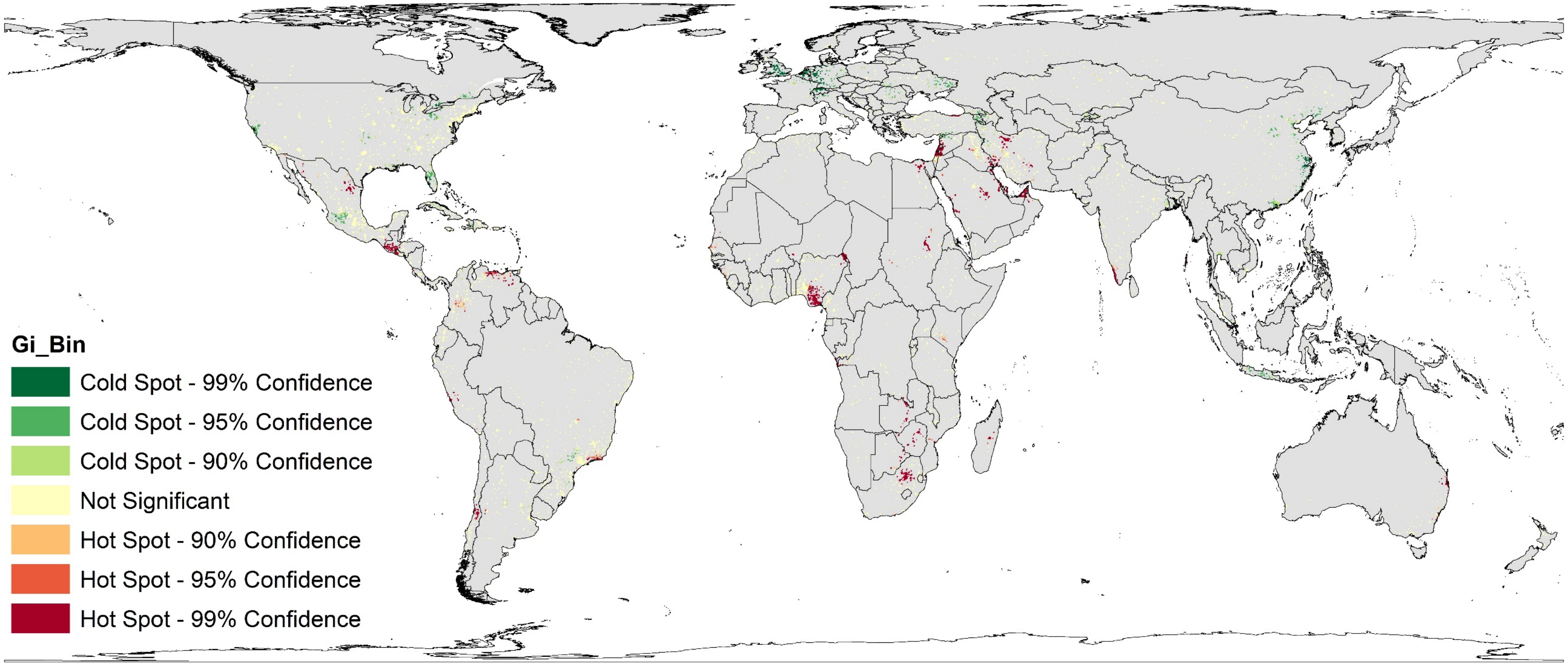

Supplementary Fig. 4 Future hot-spots and coldspots of habitat loss due to urban expansion under SSP3. The Hot Spot Analysis (Getis-Ord Gi*) tool in ArcGIS Pro 2.5 was used. This tool identifies statistically significant spatial clusters of high values (hot spots) and low values (cold spots). Potential habitat loss due to future urban expansion was input variable. The Gi_Bin identifies statistically significant hot and cold spots. Statistical significance was based on the *P*-value and *Z*-score (two-sided), and no adjustments were made for multiple comparisons.

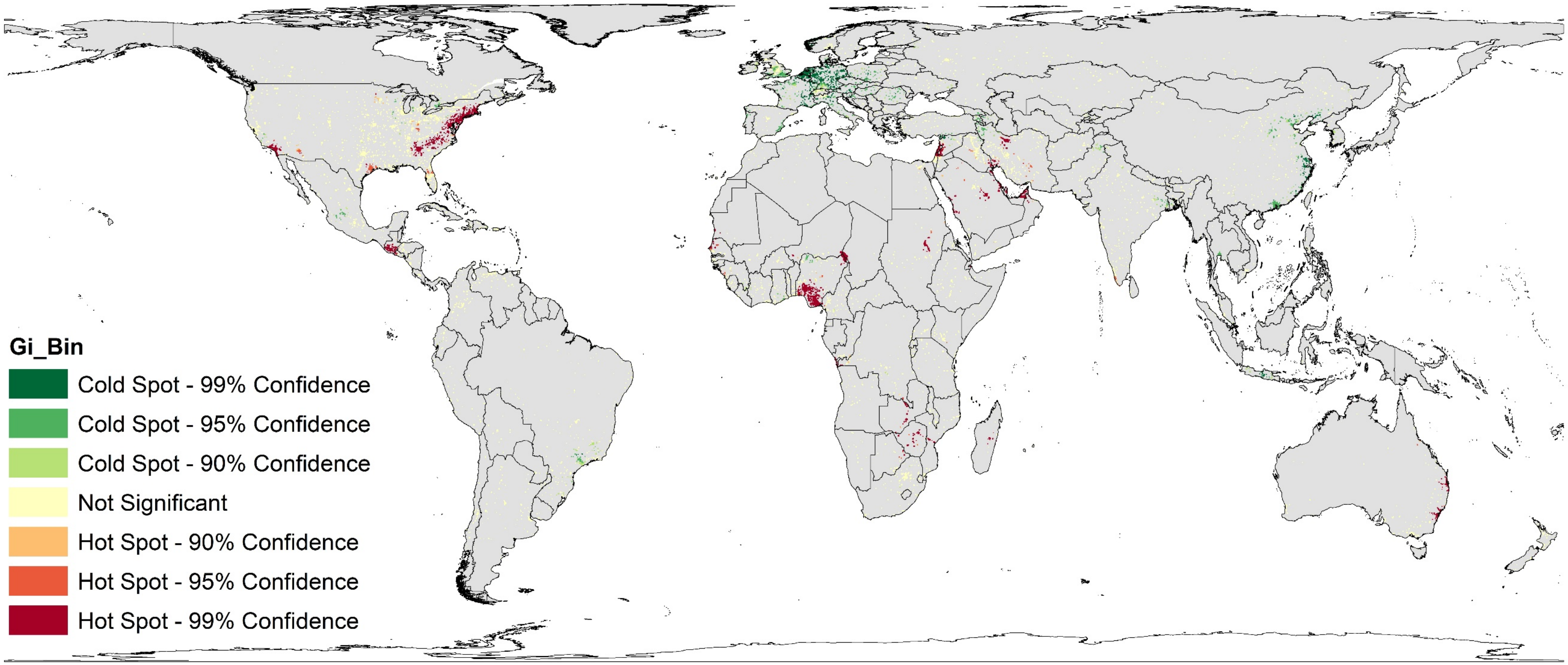

Supplementary Fig. 5 Future hot-spots and coldspots of habitat loss due to urban expansion under SSP4. The Hot Spot Analysis (Getis-Ord Gi*) tool in ArcGIS Pro 2.5 was used. This tool identifies statistically significant spatial clusters of high values (hot spots) and low values (cold spots). Potential habitat loss due to future urban expansion was input variable. The Gi_Bin identifies statistically significant hot and cold spots. Statistical significance was based on the *P*-value and *Z*-score (two-sided), and no adjustments were made for multiple comparisons.

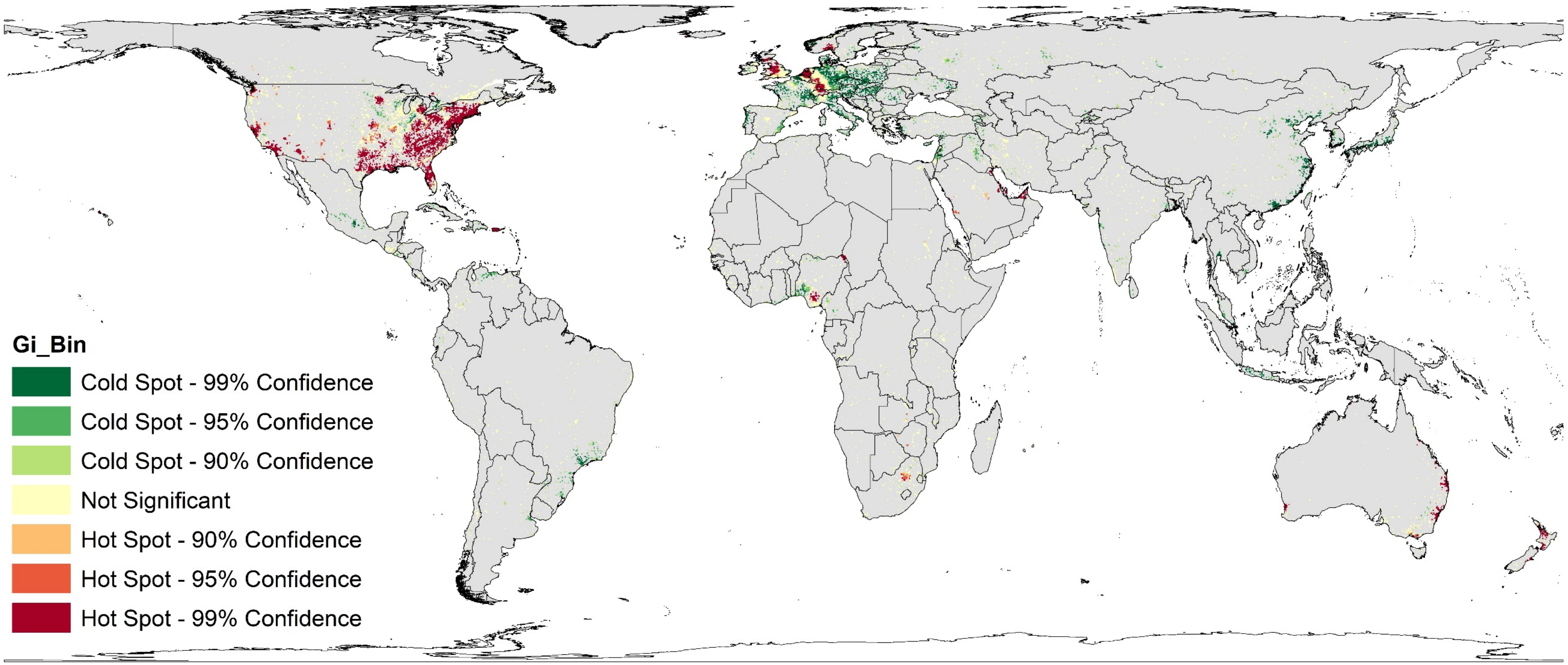

Supplementary Fig. 6 Future hot-spots and coldspots of habitat loss due to urban expansion under SSP5. The Hot Spot Analysis (Getis-Ord Gi*) tool in ArcGIS Pro 2.5 was used. This tool identifies statistically significant spatial clusters of high values (hot spots) and low values (cold spots). Potential habitat loss due to future urban expansion was input variable. The Gi_Bin identifies statistically significant hot and cold spots. Statistical significance was based on the *P*-value and *Z*-score (two-sided), and no adjustments were made for multiple comparisons.

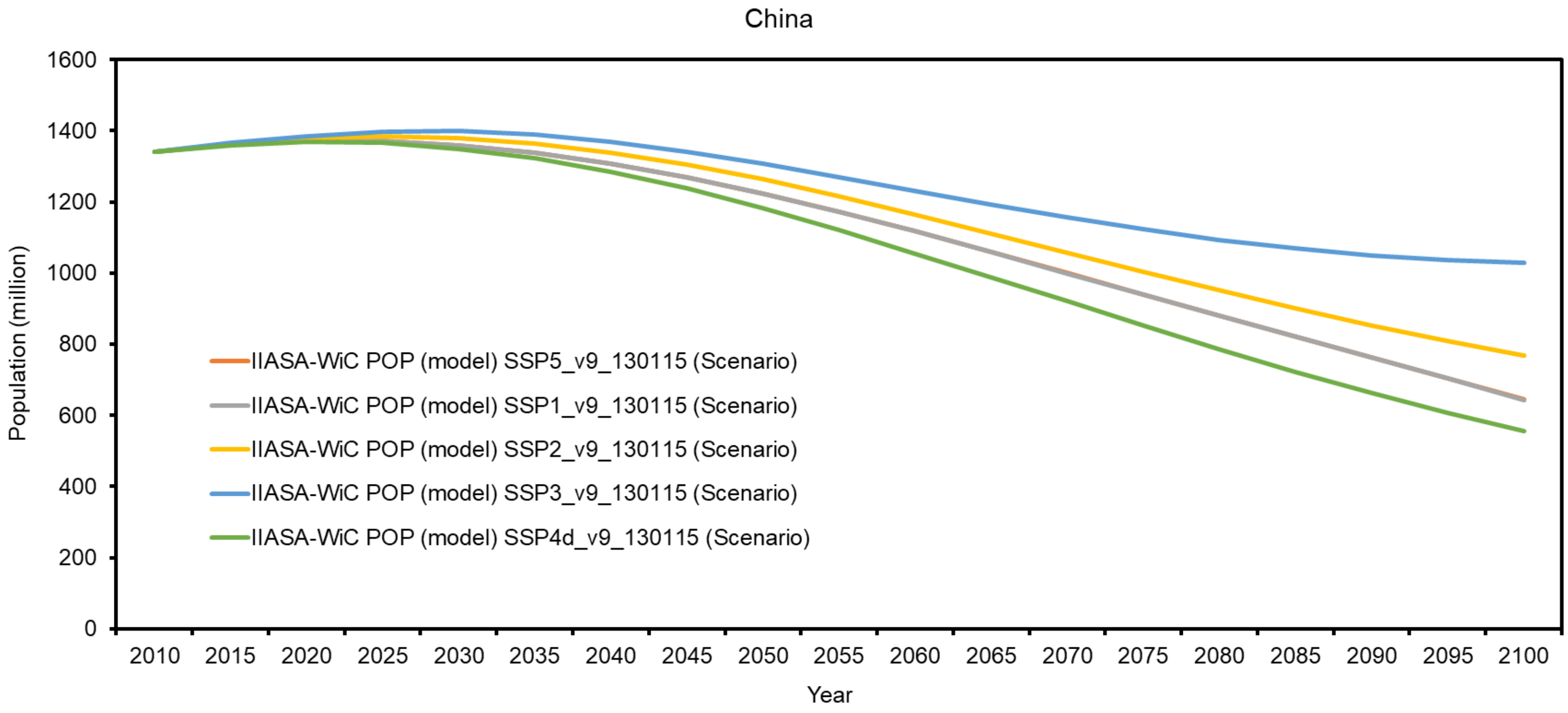

Supplementary Fig. 7 The changes in total population (in million) in China from 2010 to 2100 based on the SSP Database version 2.0.

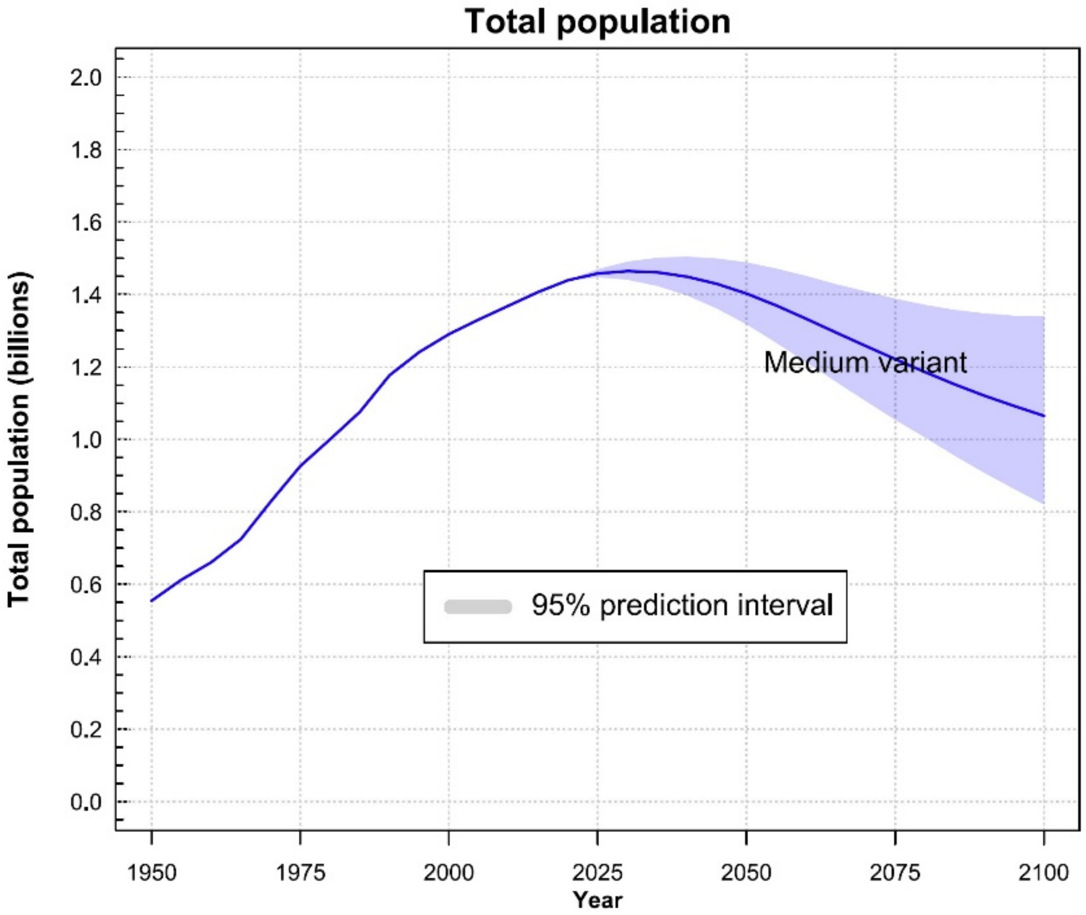

Supplementary Fig. 8 The changes in total population (in billions) in China from 1950 to 2100 based on

the World Population Prospects 2019, UN.

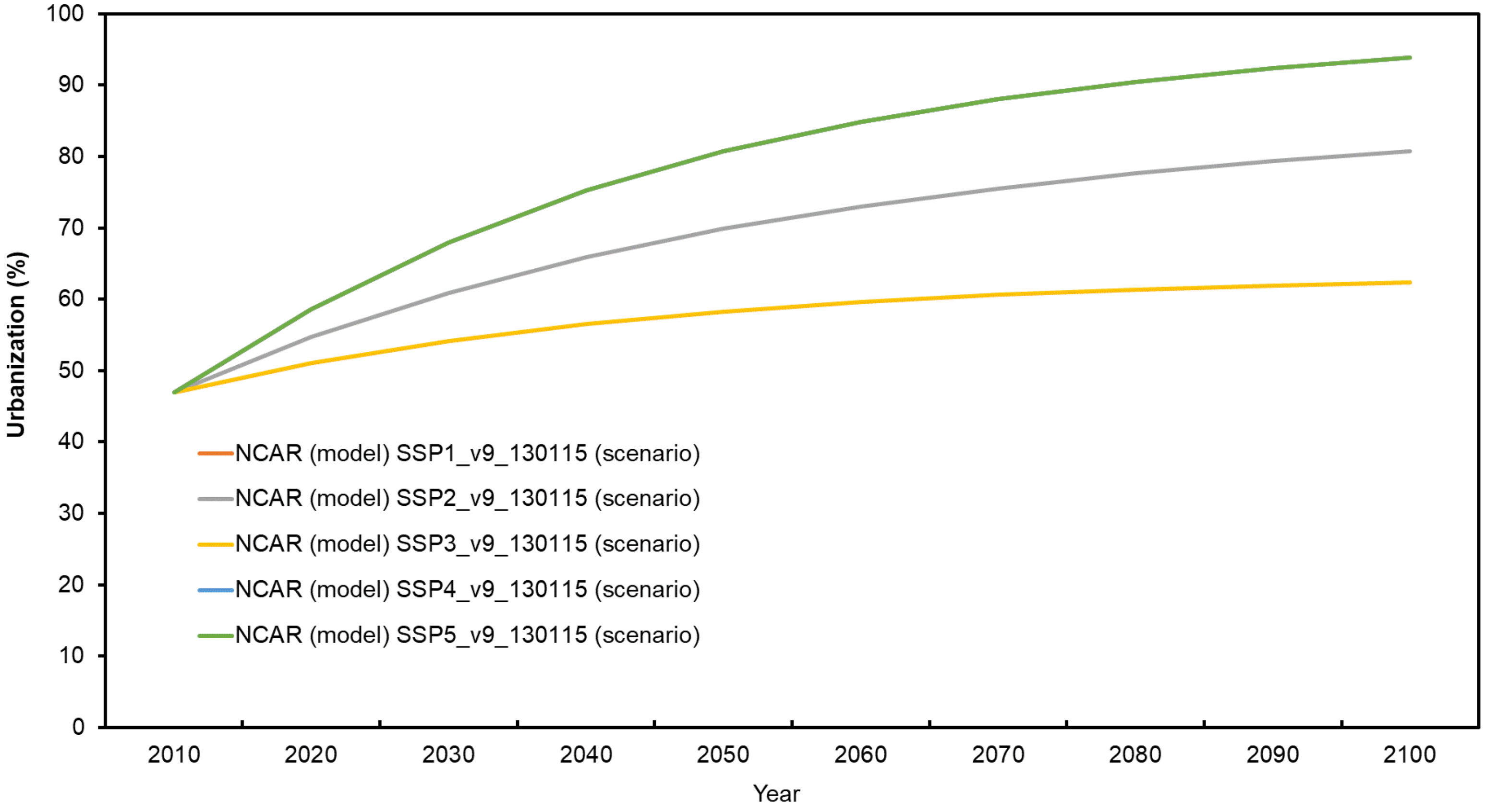

Supplementary Fig. 9 Future urbanization estimation in China from 2010 to 2100 based on the SSP Database version 2.0.

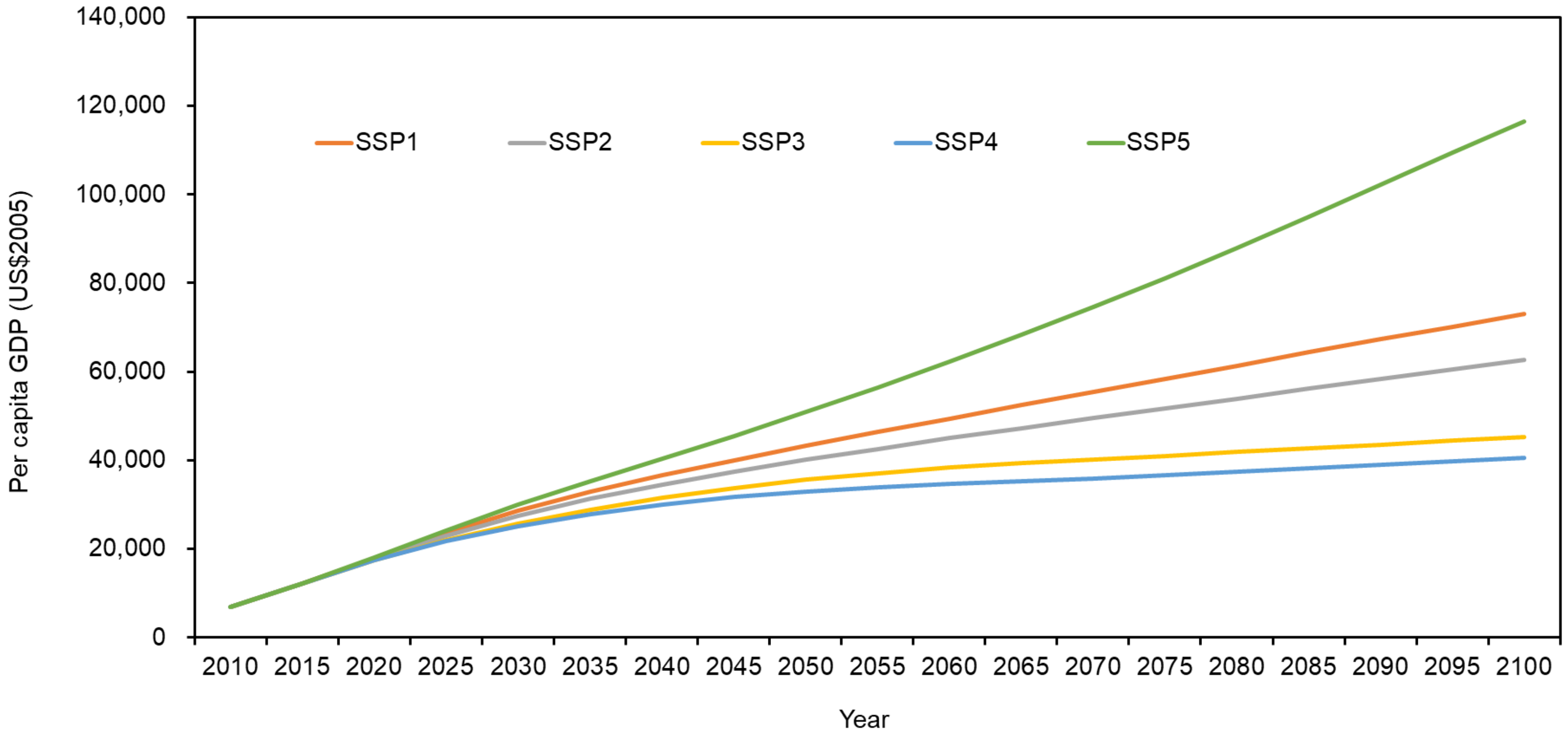

Supplementary Fig. 10 Future per capita GDP estimation in China from 2010 to 2100 based on the SSP Database version 2.0.

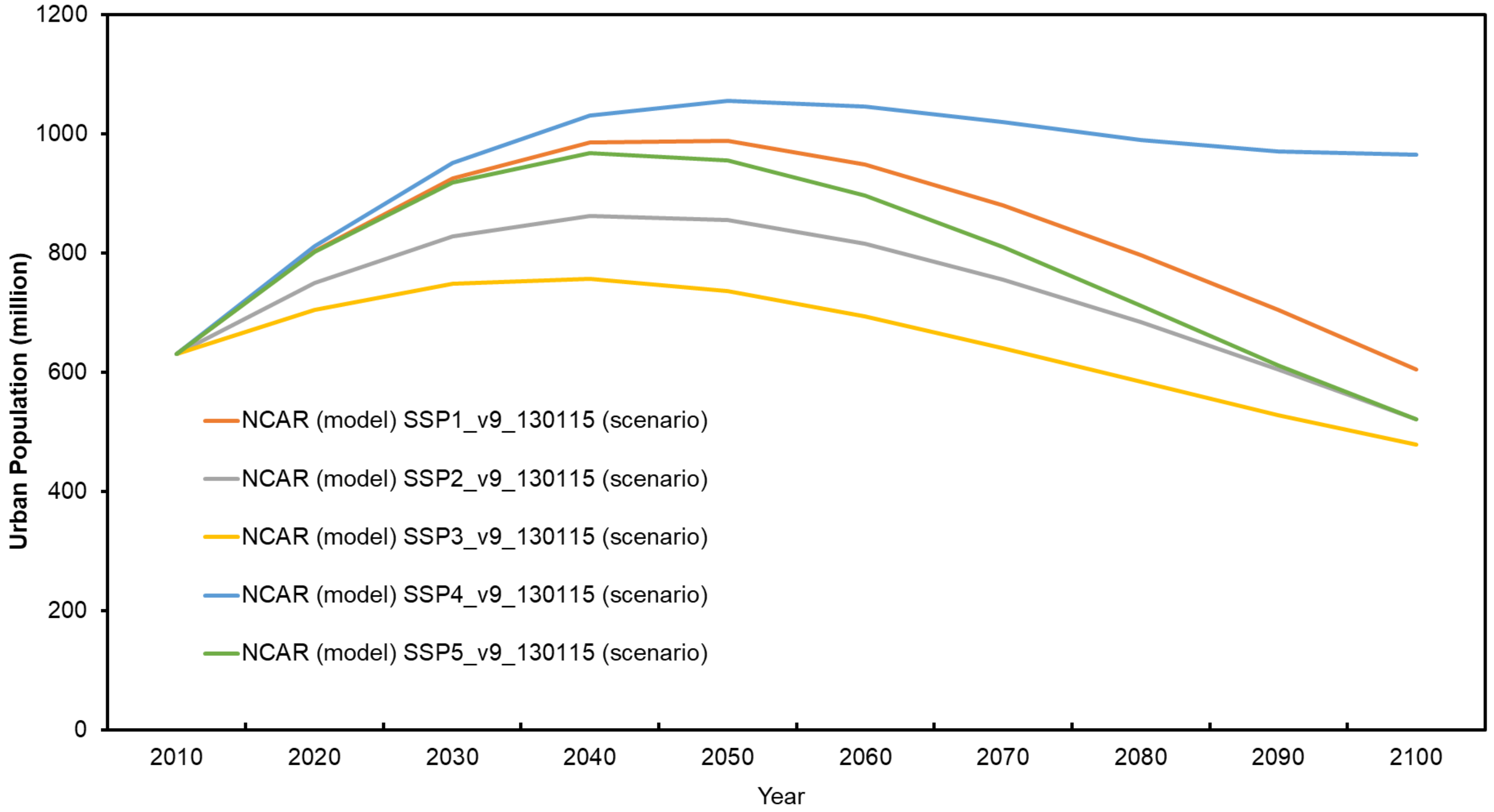

Supplementary Fig. 11 Future urban population estimation in China from 2010 to 2100 based on the SSP Database version 2.0.

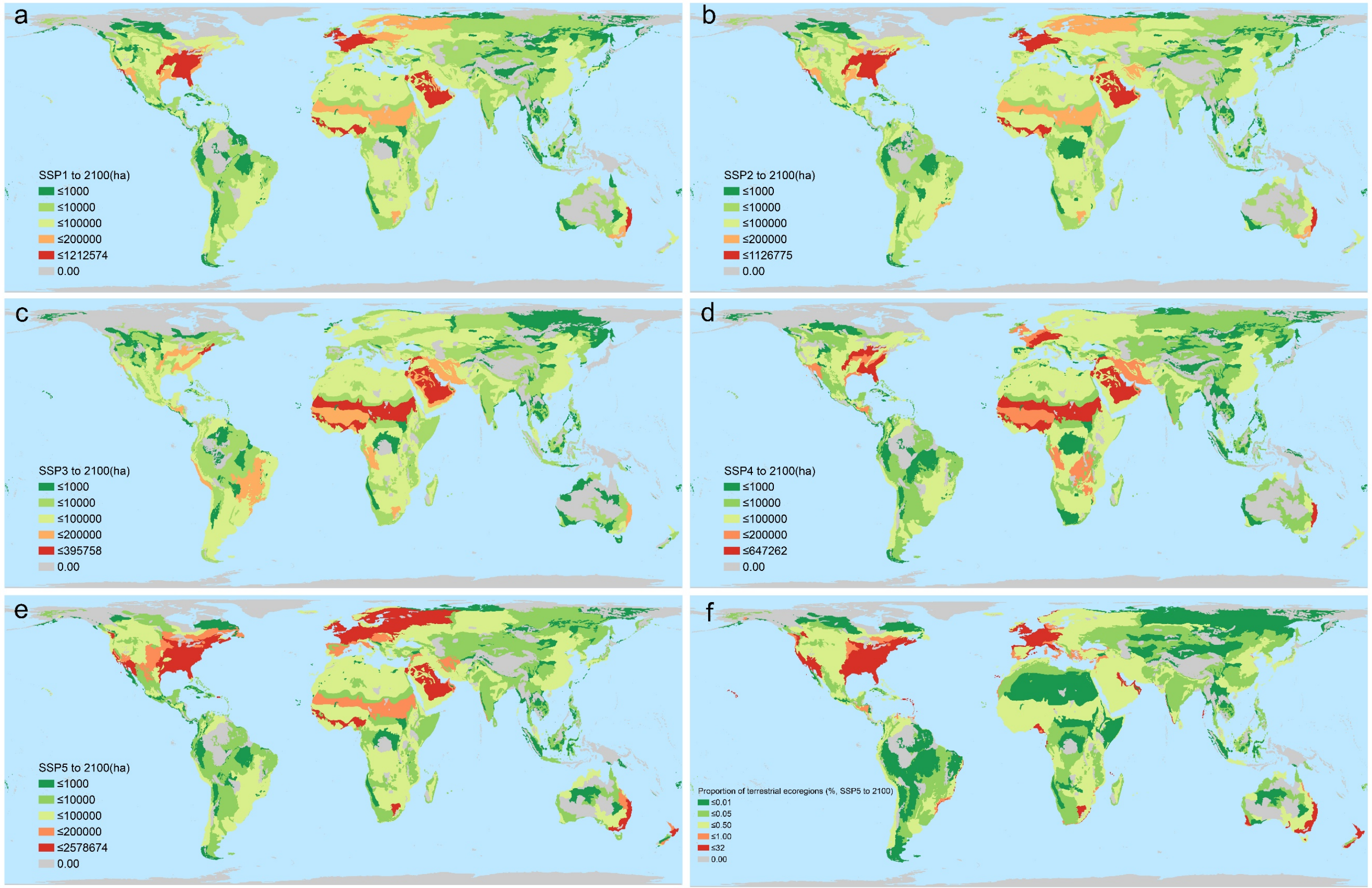

Supplementary Fig. 12 Future habitat loss due to urban expansion at the ecoregional scale under SSP scenarios by 2100. (a) the ecoregional statistics of habitat loss area caused by urban expansion under SSP1 scenario; (b) under SSP2 scenario; (c) under SSP3 scenario; (d) under SSP4 scenario; (e) under SSP5 scenario; (f) future habitat loss proportion at the ecoregional level caused by urban expansion under SSP5.

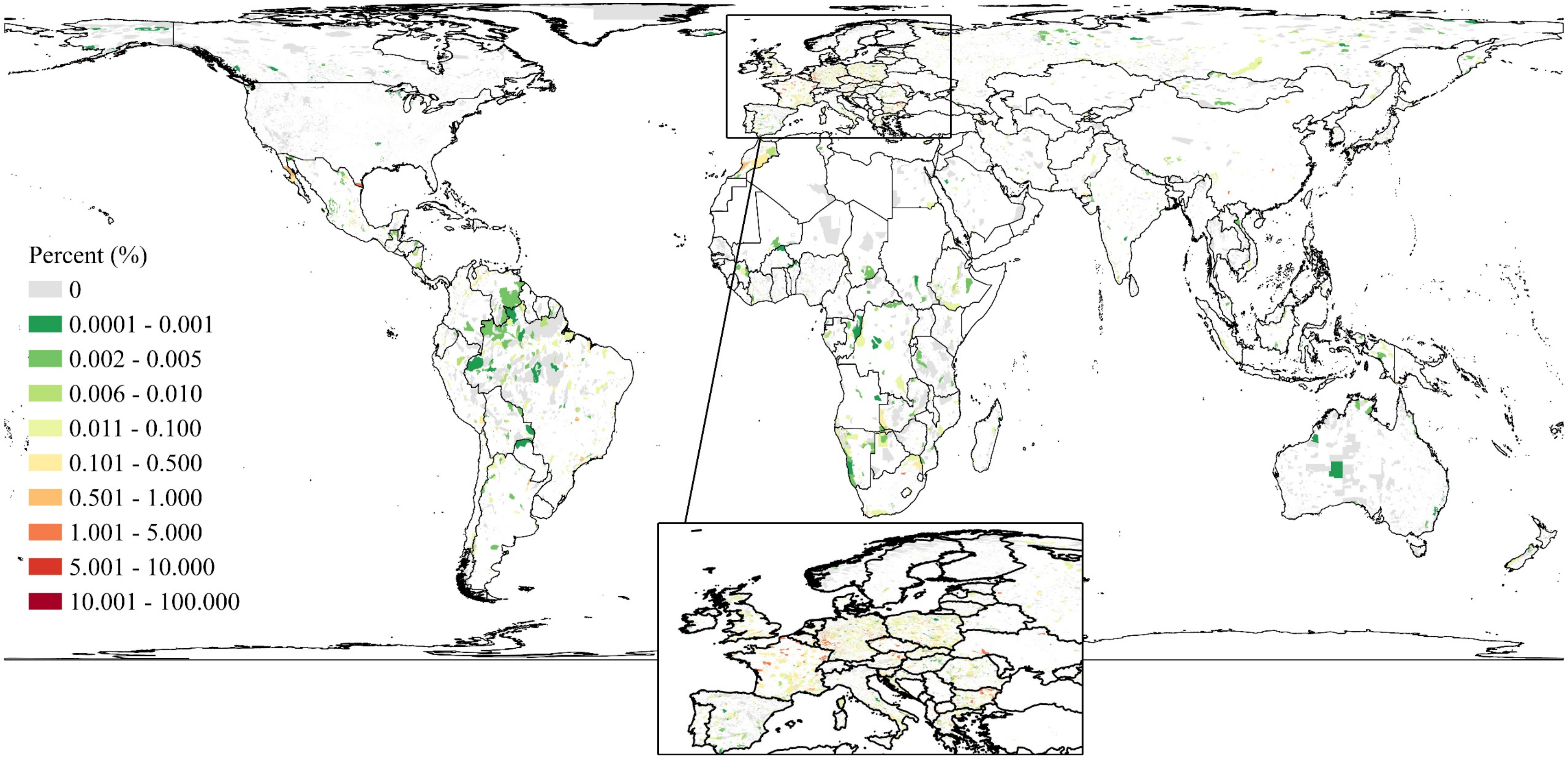

Supplementary Fig. 13 The precent of urban land within protected areas in 1992. The urban area within PAs was 8,290 km^2 in 1992. The number of protected areas affected by urban land was 21,217. The number and total area of protected areas with IUCN categories I and II were 558 and 579.31 km^2, respectively. These results were based on global LC 1992 map produced by the European Space Agency (ESA) Climate Change Initiative (CCI) with a resolution of 300 meters.

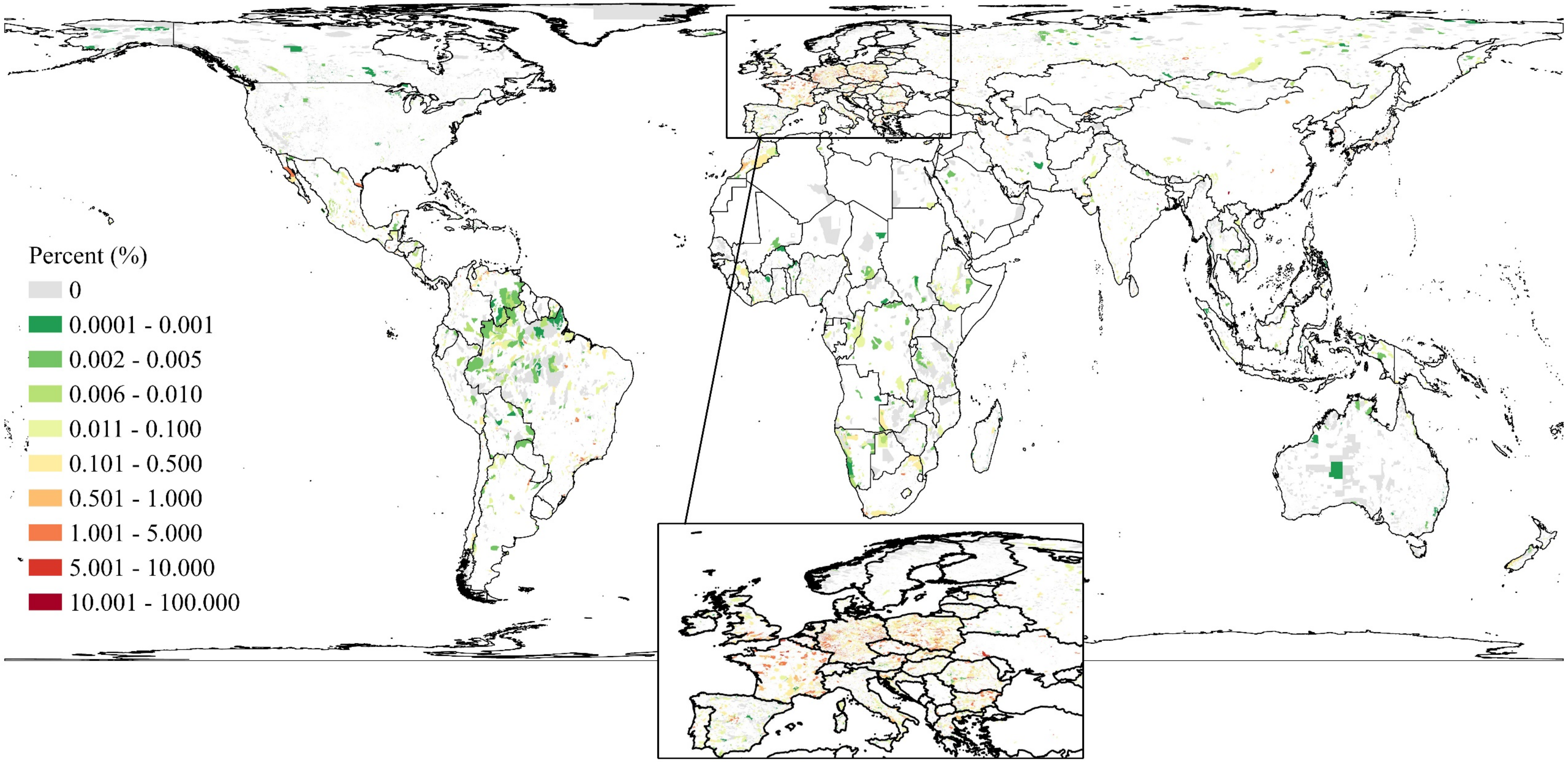

Supplementary Fig. 14 The precent of urban land within protected areas in 2015. The urban area within PAs was 20,625 km^2 in 2015. The number of protected areas affected by urban land was 35,161. The number and total area of protected areas with IUCN categories I and II were 813 and 1229.95 km^2, respectively. These results were based on global LC 2015 map produced by the European Space Agency (ESA) Climate Change Initiative (CCI) with a resolution of 300 meters.

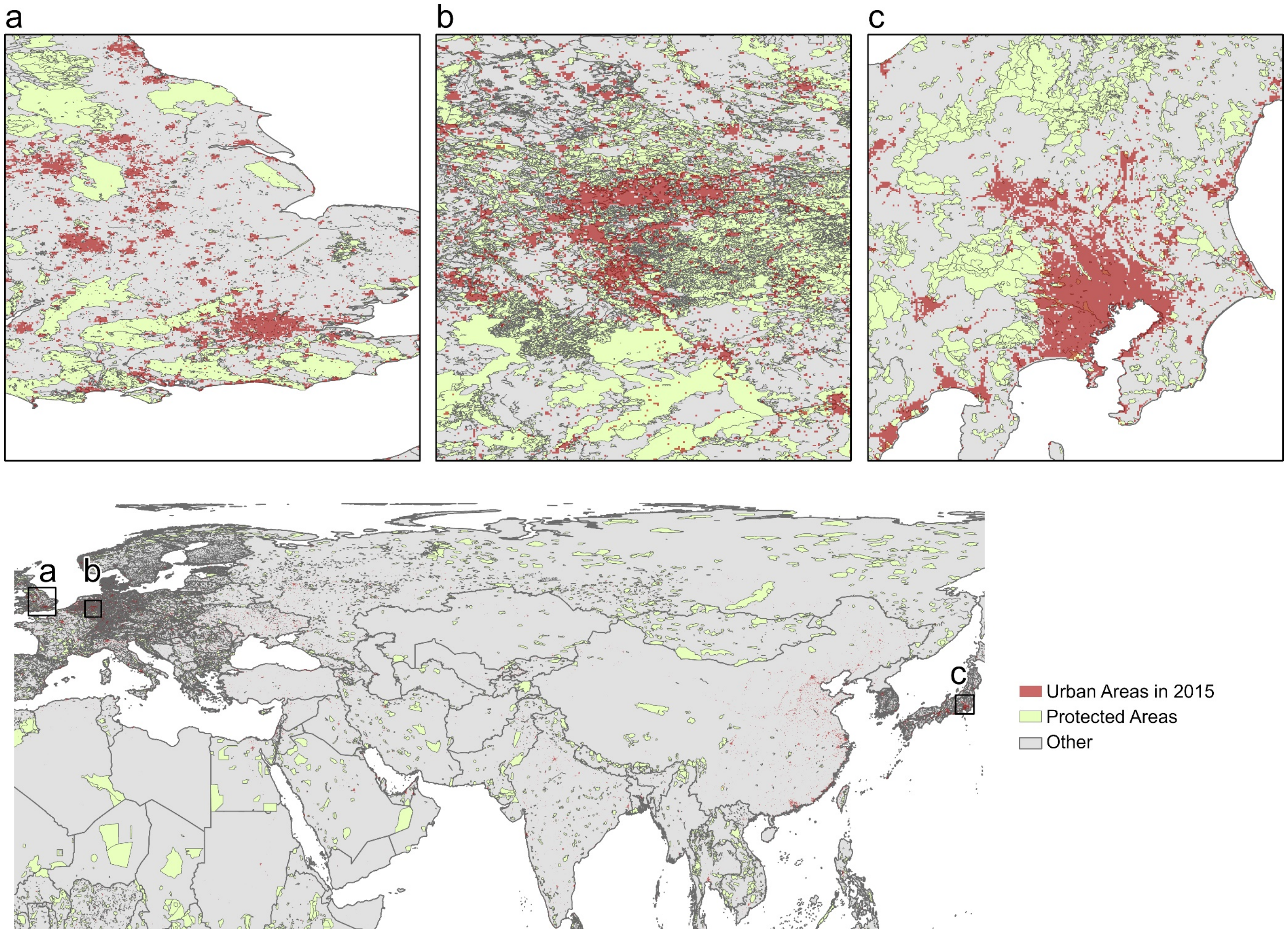

Supplementary Fig. 15 Urban areas that were distributed in protected areas in 2015.

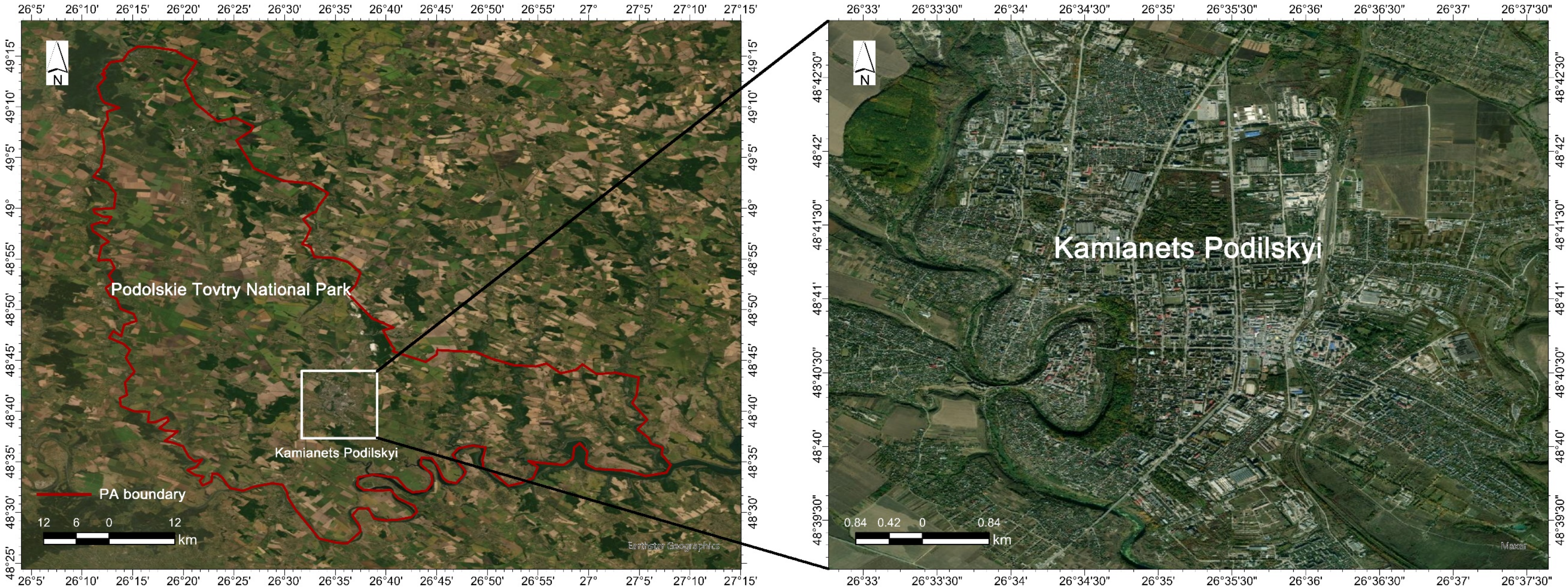

Supplementary Fig. 16 Kamianets-Podilskyi, a city within Podolskie Tovtry National Park, Ukraine.

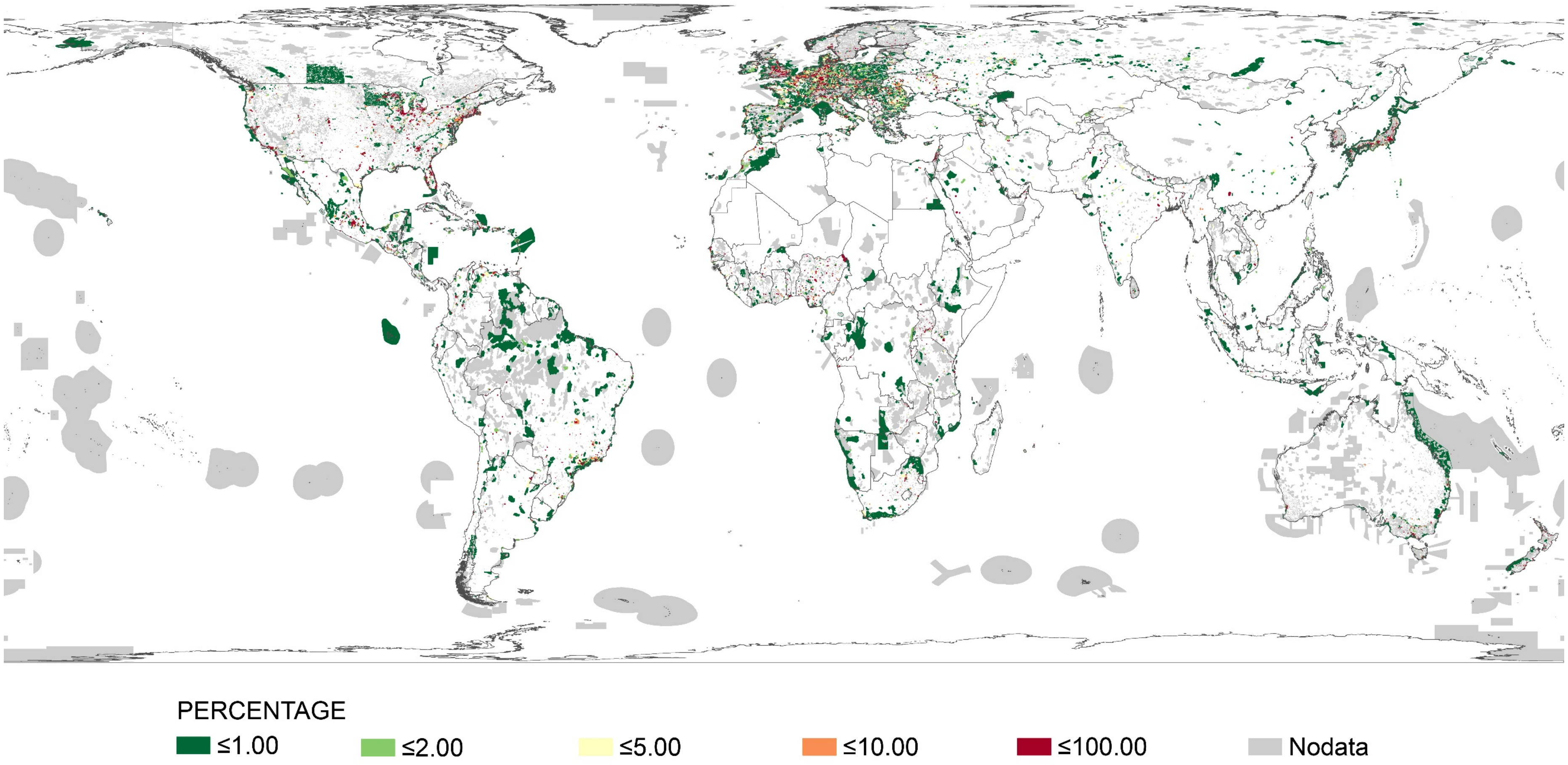

Supplementary Fig. 17 The proportion of urban land in each protected area by 2100 under SSP3 scenario.

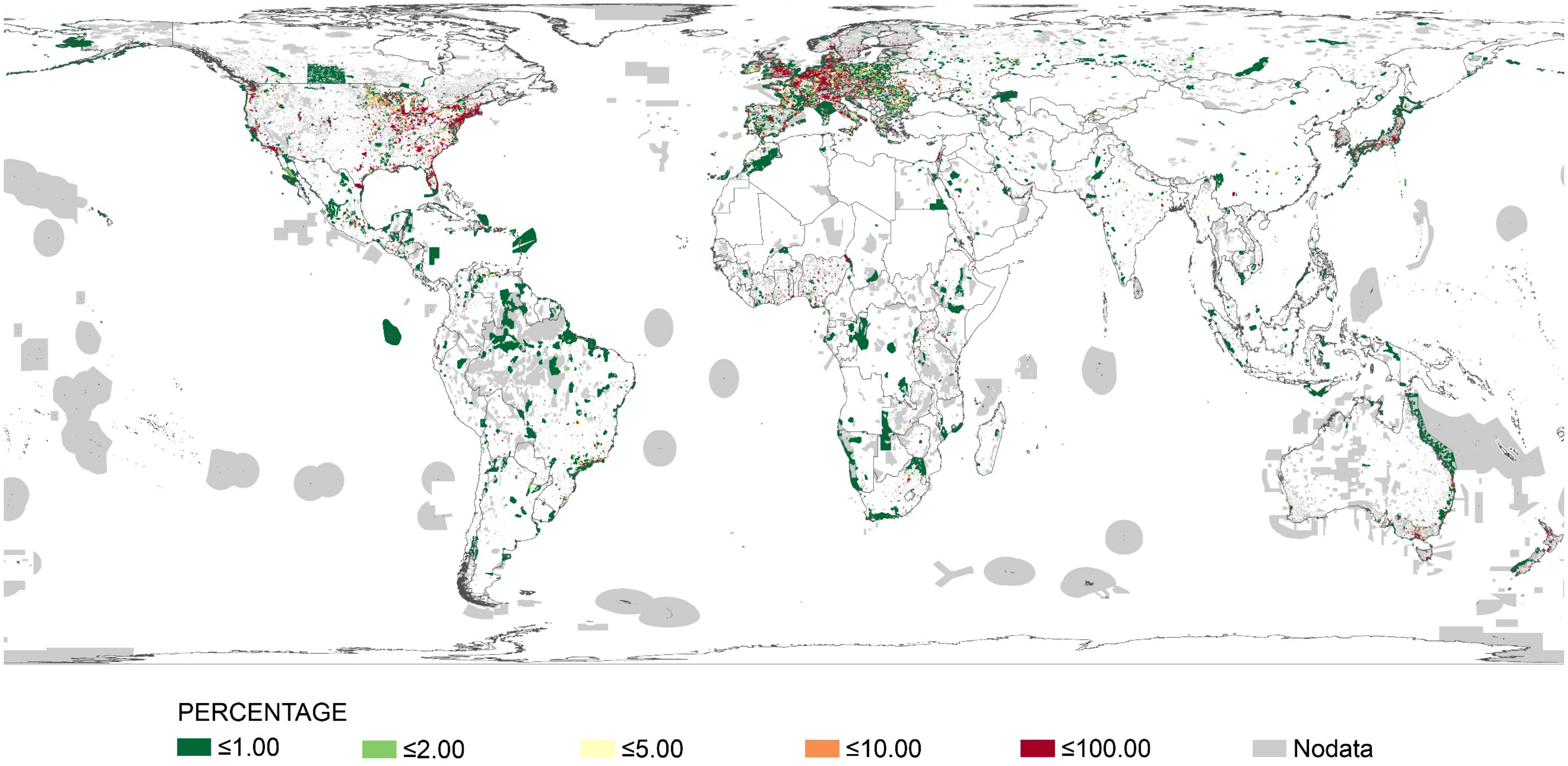

Supplementary Fig. 18 The proportion of urban land in each protected area by 2100 under SSP5 scenario.

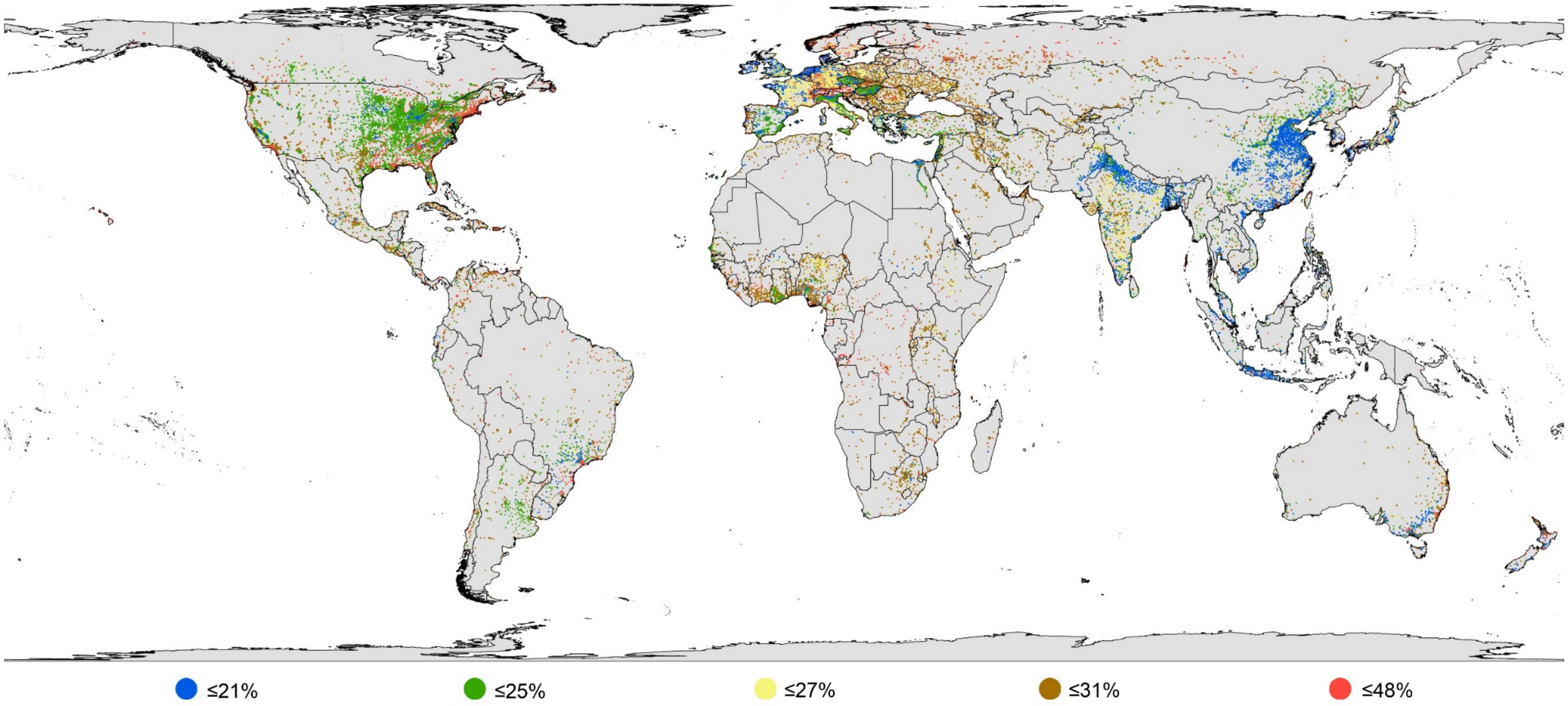

Supplementary Fig. 19 Future overall relative species richness losses due to urban expansion under SSP5 scenario in 1-km grid.

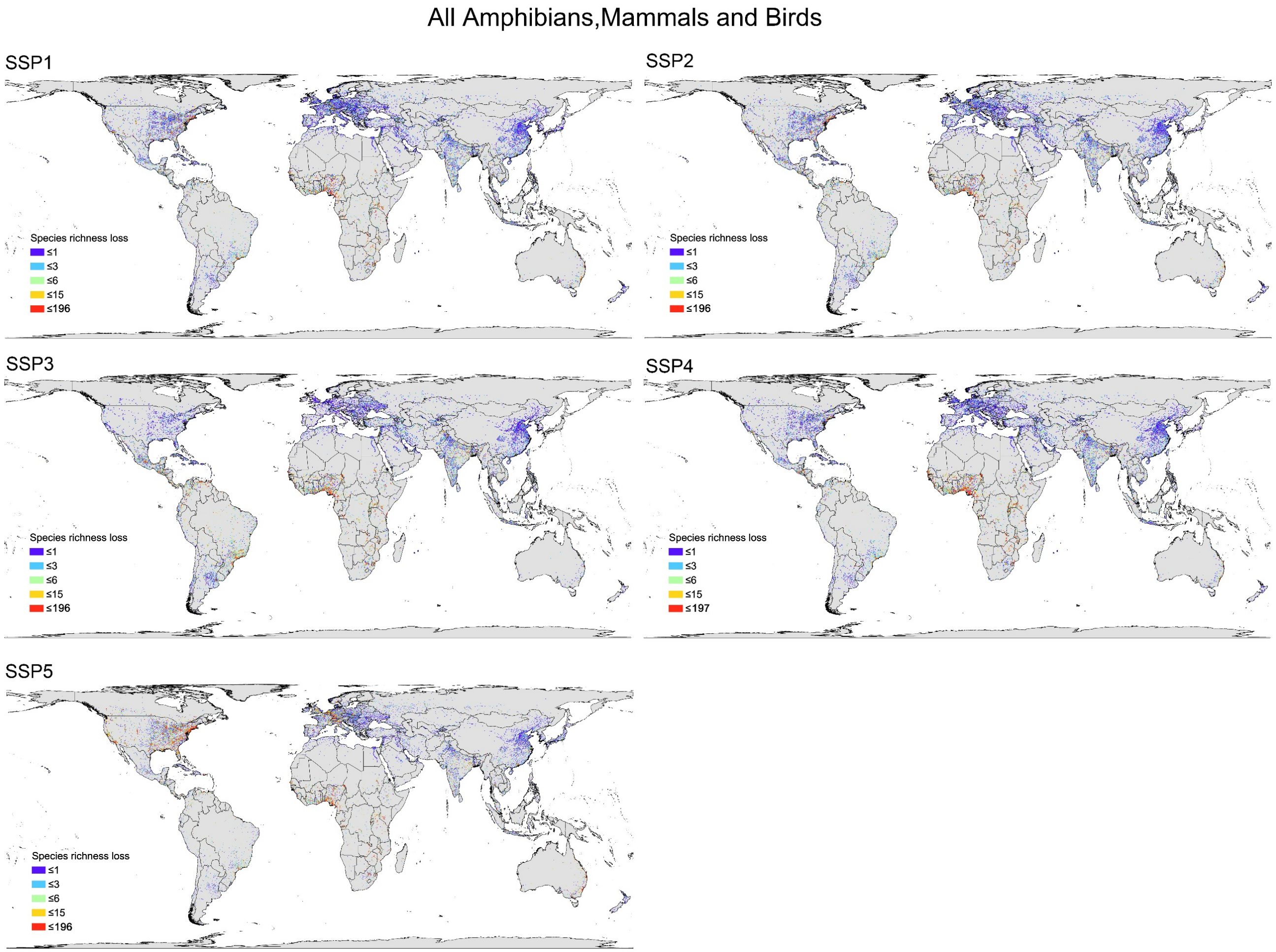

Supplementary Fig. 20 Future potential species richness loss of all amphibians, mammals and birds due to urban expansion under SSP scenarios in 10-km grid.

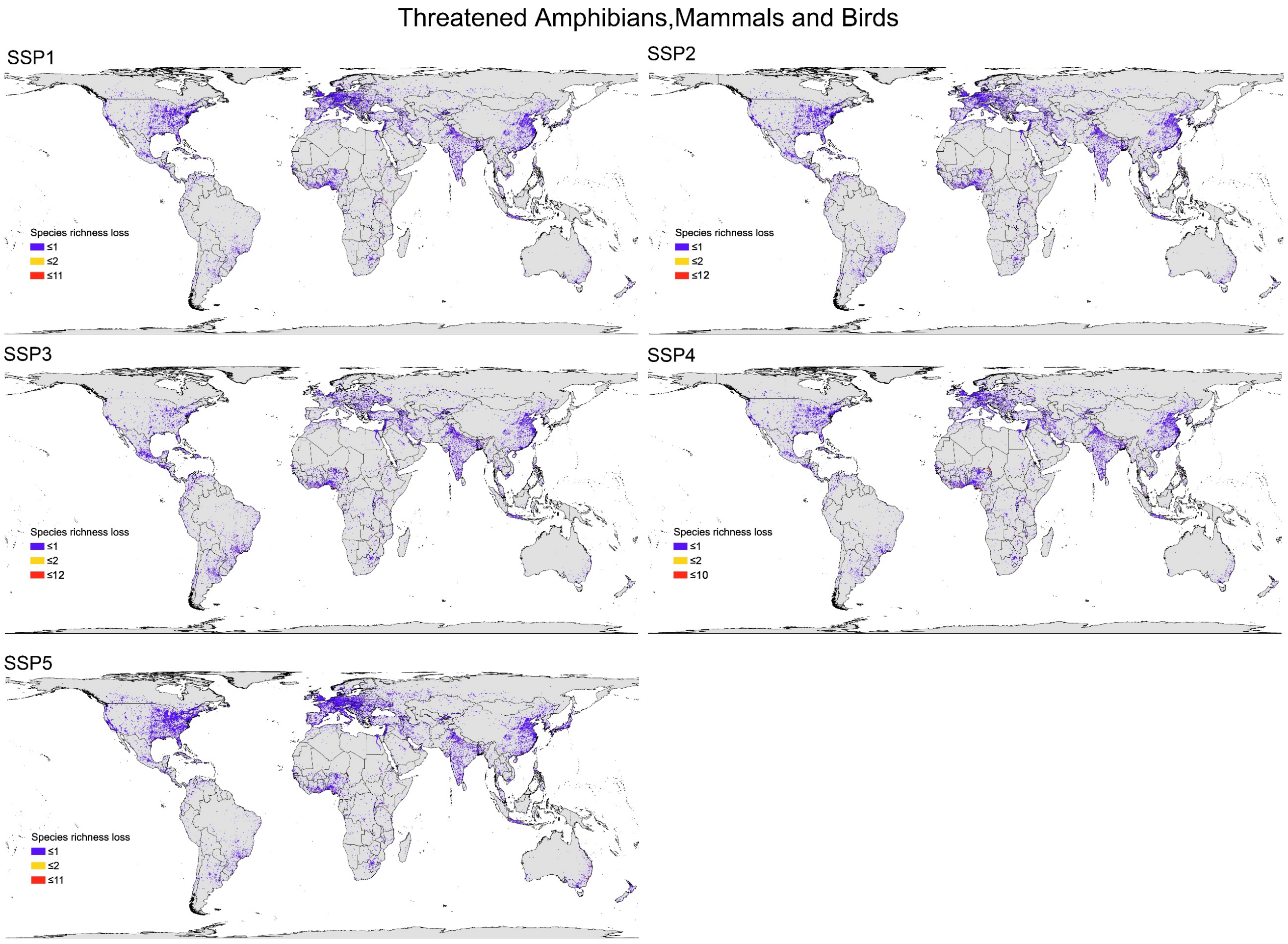

Supplementary Fig. 21 Future potential species richness loss of threatened amphibians, mammals and birds due to urban expansion under SSP scenarios in 10-km grid.

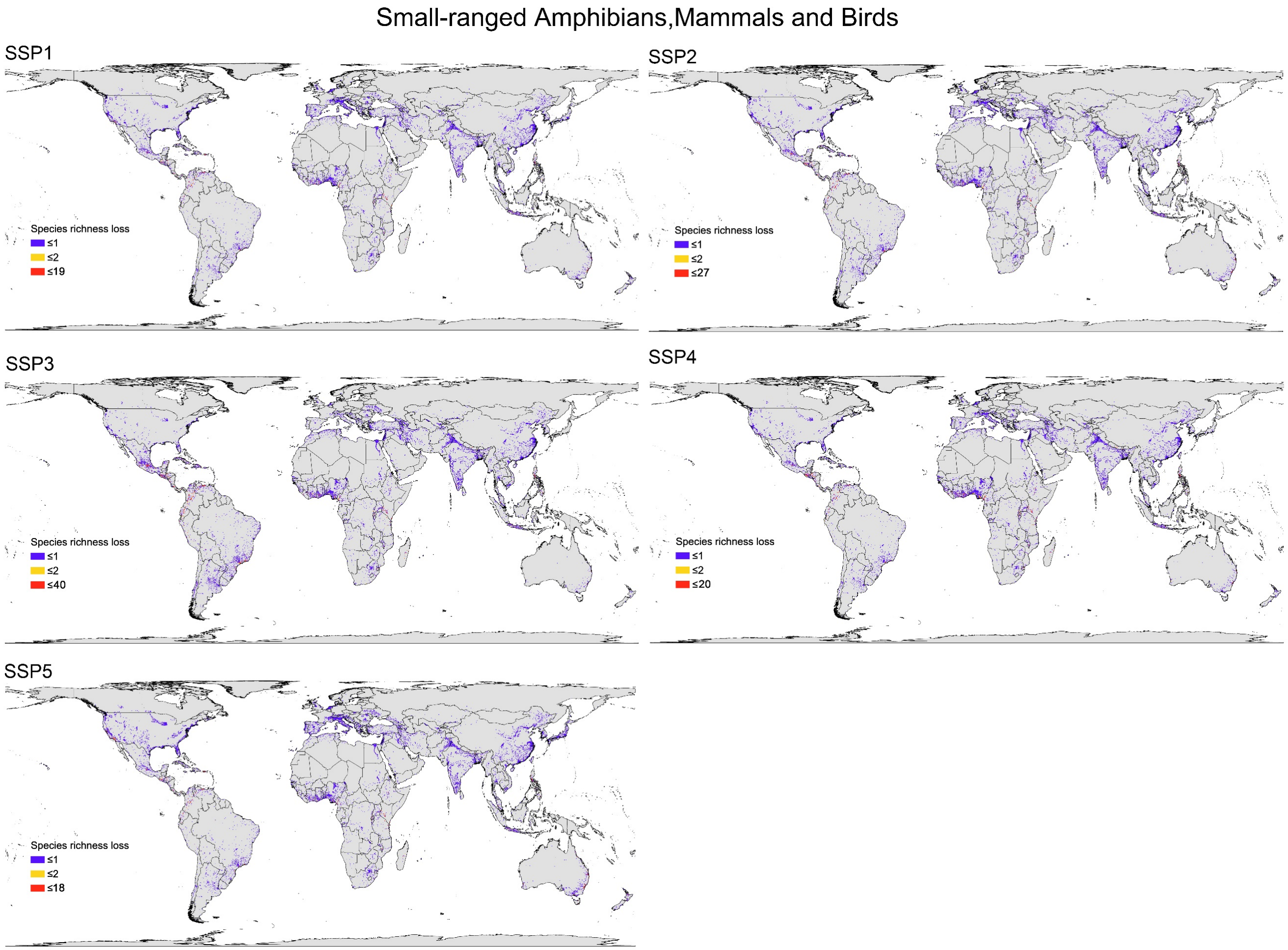

Supplementary Fig. 22 Future potential species richness loss of small-ranged amphibians, mammals and birds due to urban expansion under SSP scenarios in 10-km grid.

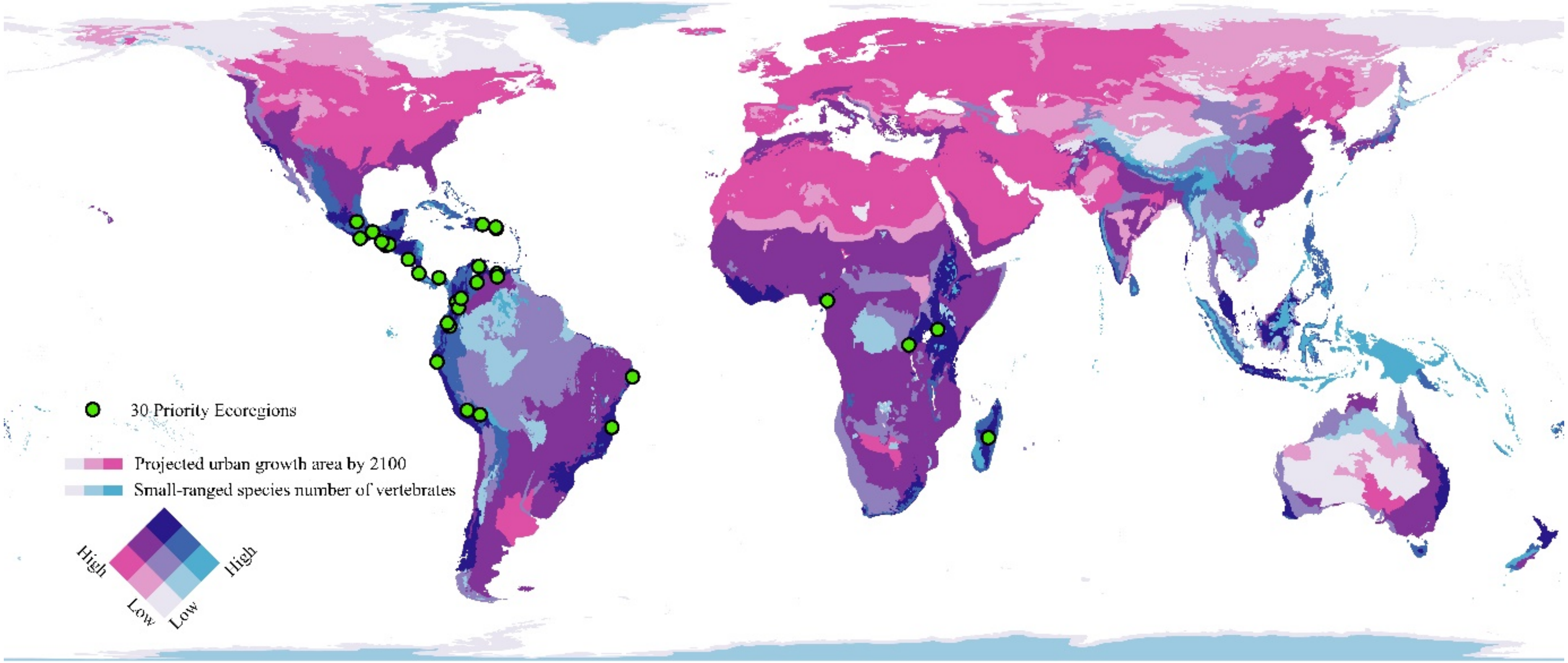

Supplementary Fig. 23 Conservation-priority ecoregions for species loss caused by future urban growth (mean of five SSPs).

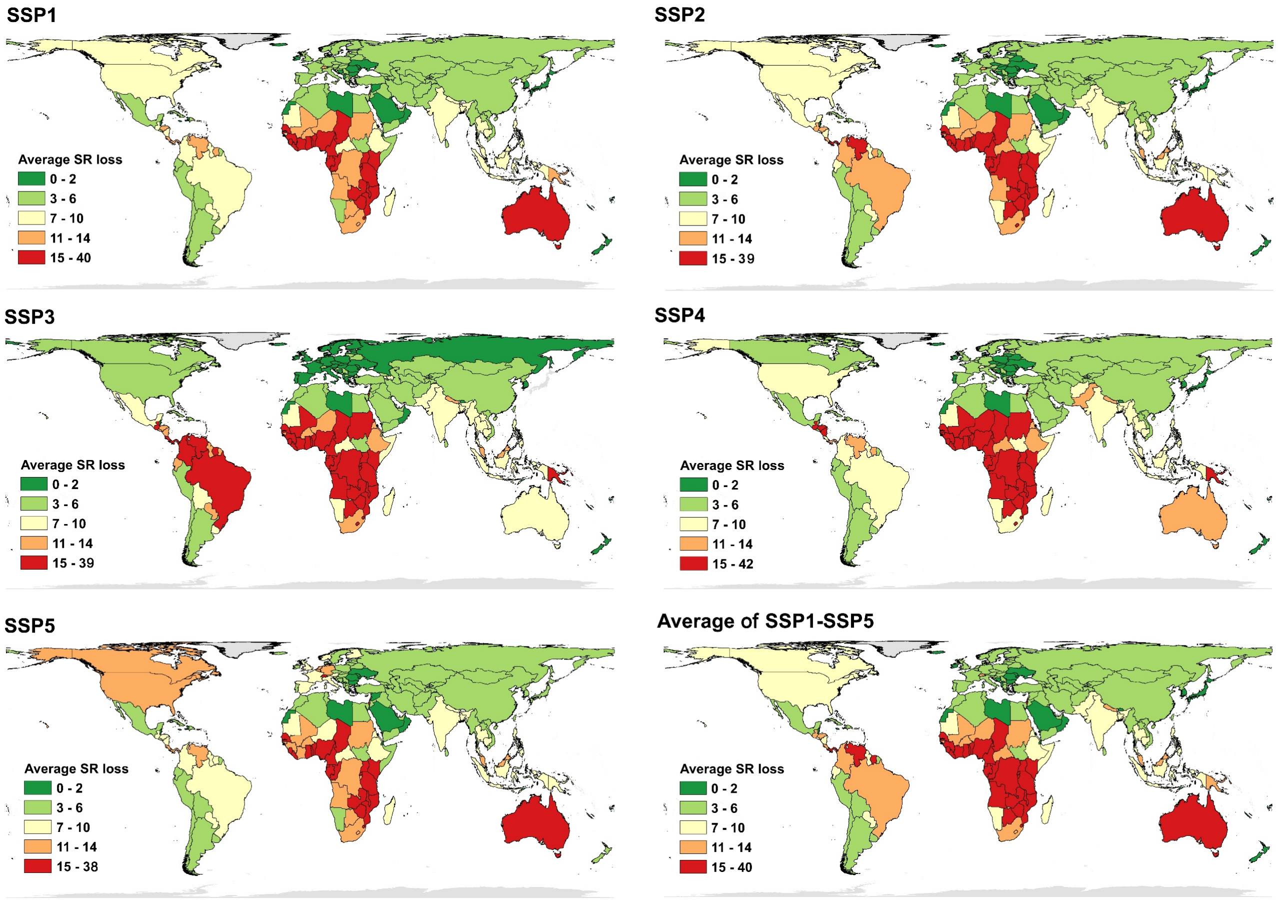

Supplementary Fig. 24 National average potential biodiversity loss per 10-km grid cell due to future urban expansion under SSP scenarios. The national mean potential biodiversity loss in terms of average number of terrestrial vertebrate species (amphibians, mammals, and birds) lost per 10-km grid cell. SR= Species Richness. Gray areas were not considered in this analysis.

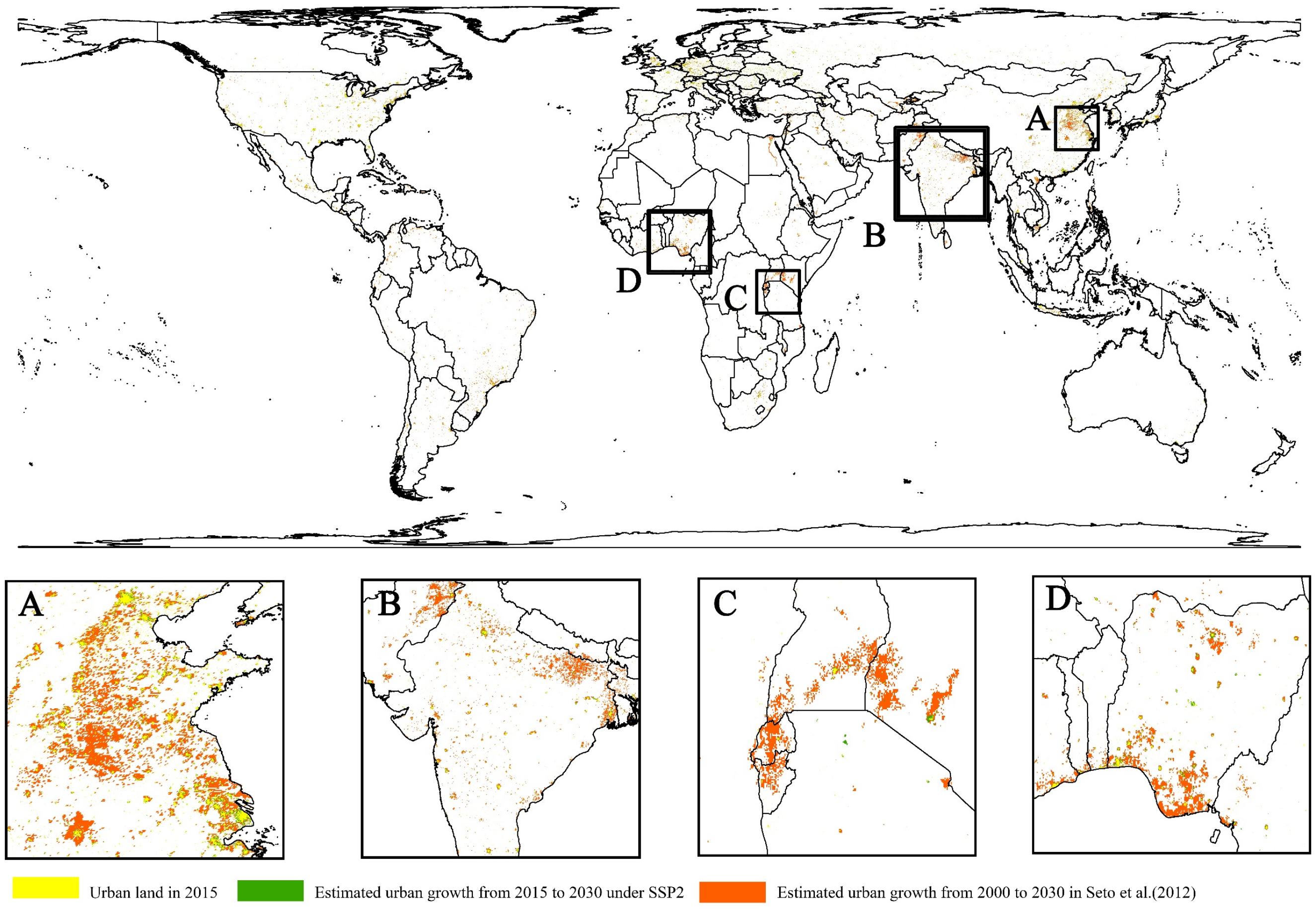

Supplementary Fig. 25 Global comparison between urban expansion forecasts of Seto et al. (2012) and our projection results under SSP2 (based on Chen et al., 2020). (**A**) China, (**B**) India, (**C**) East Africa, and (**D**) West Africa.

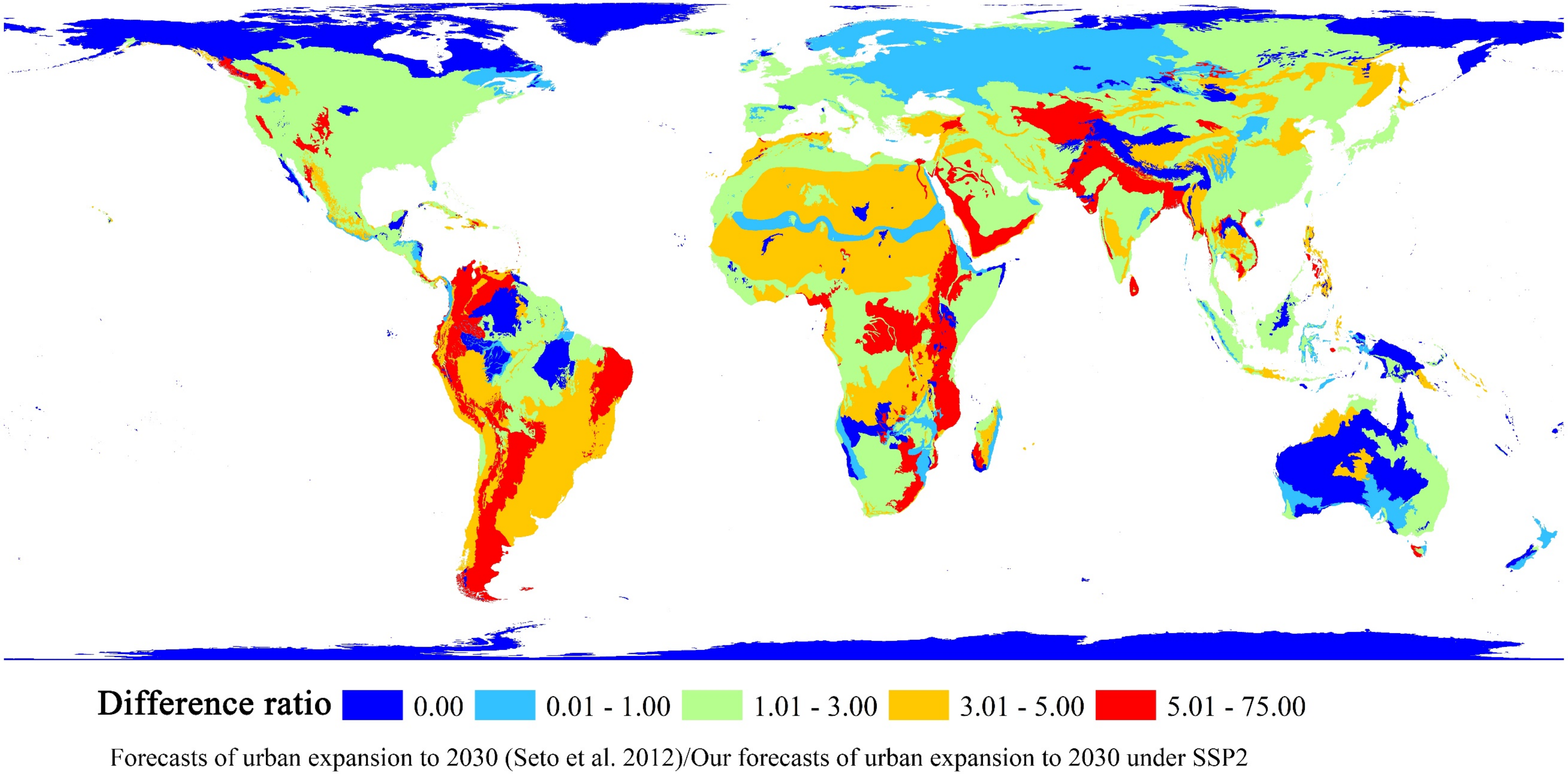

Supplementary Fig. 26 Difference ratio of urban expansion forecasts between Seto et al. 2012 and our SSP2 projection results (based on Chen et al., 2020).

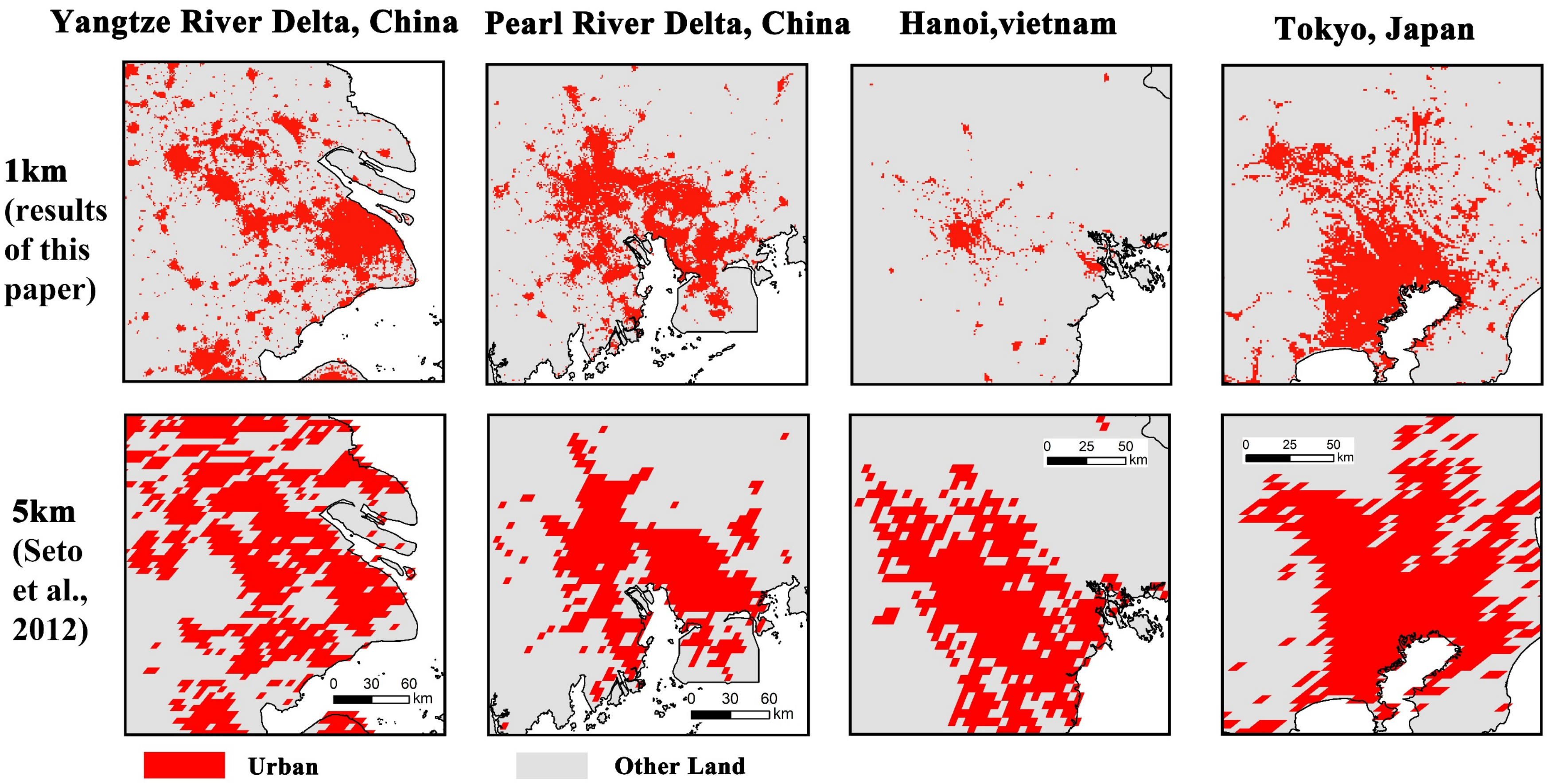

Supplementary Fig. 27 Differences in urban details for some metropolitan areas around the world for the year 2030 using 1-km resolution (our results based on Chen et al., 2020) and 5-km resolution (Seto et al., 2012).

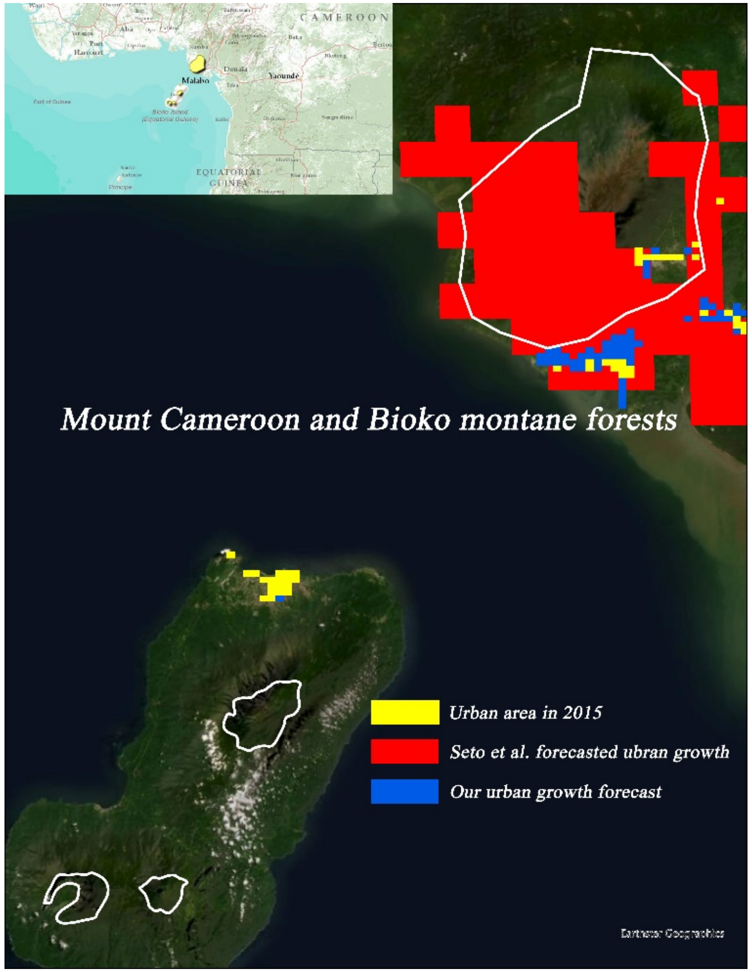

Supplementary Fig. 28 The difference in projected urban growth by Seto et al. (2012) and ours (based on Chen et al., 2020) in the Mount Cameroon and Bioko montane forests. White lines indicate the boundary of the ecoregion.

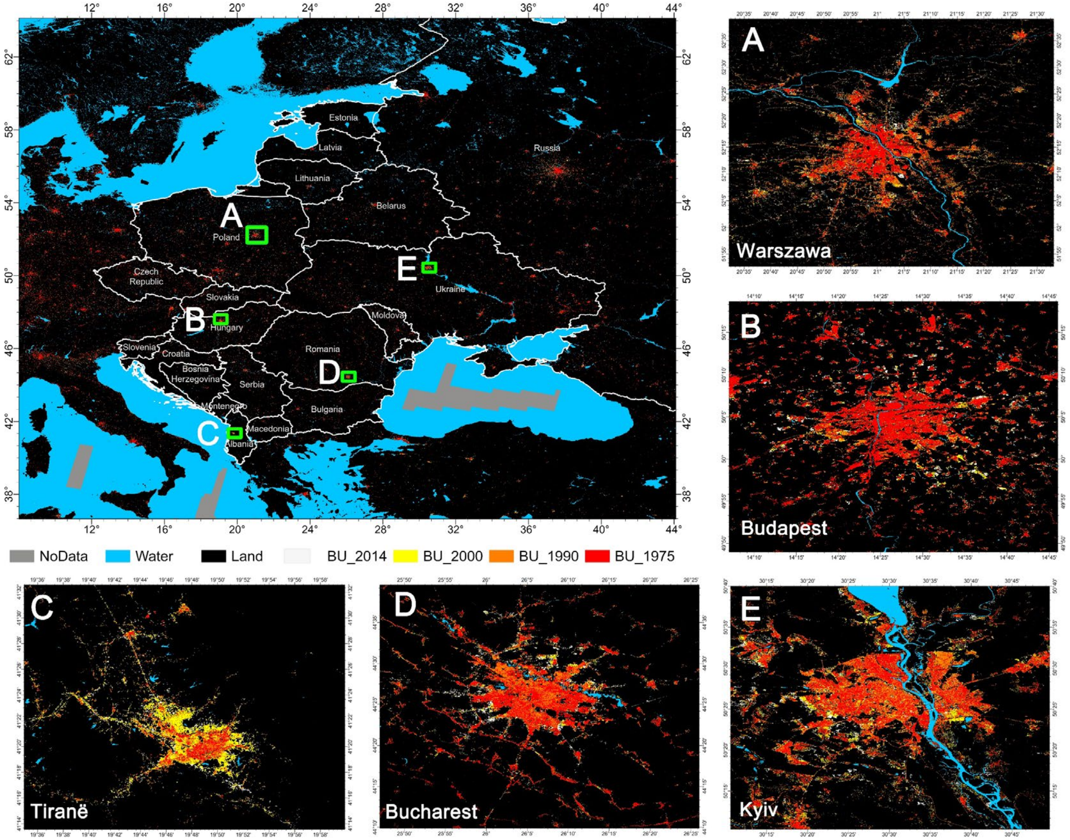

Supplementary Fig. 29 Human settlement expansion in the cities of Eastern Europe from 1975 to 2014. Data derived from the global human settlement layer (GHSL).

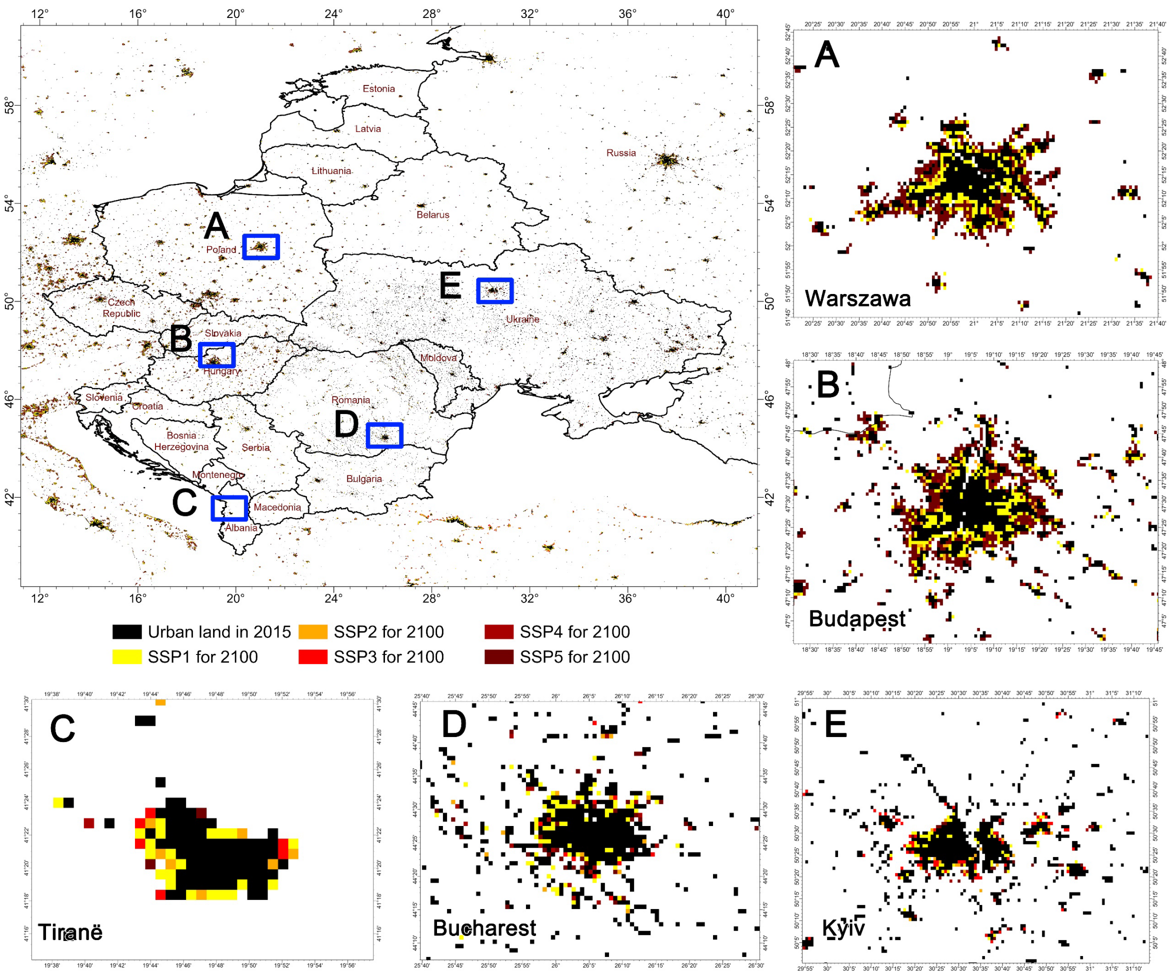

Supplementary Fig. 30 Future urban expansion in the cities of Eastern Europe from 2015 to 2100 across SSPs. These results are based on Chen et al., 2020.

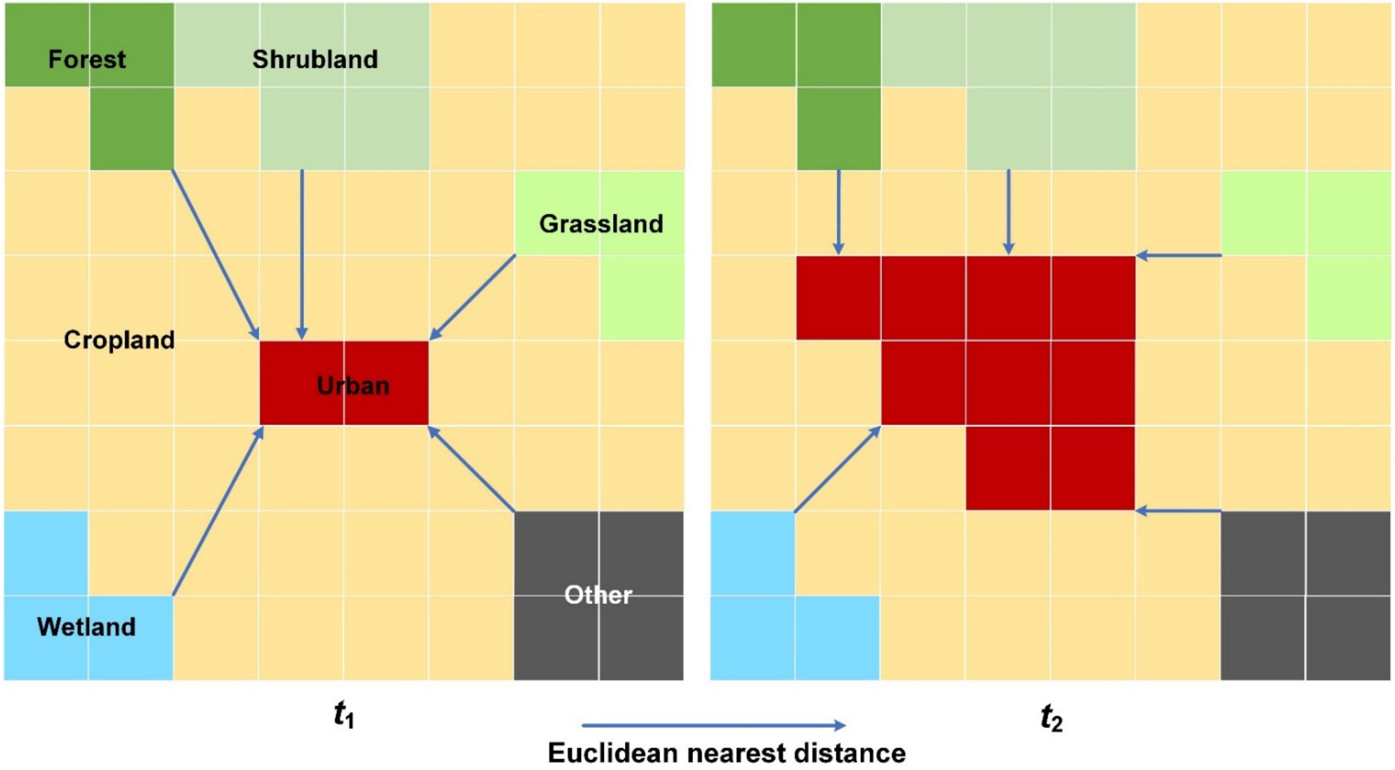

Supplementary Fig. 31 Illustration of changes in the Euclidean nearest distance between urban areas and natural habitat due to future urban expansion. Euclidean nearest distance between urban land and natural habitat gradually decreases from t_1 to t_2 due to rapid urban expansion.

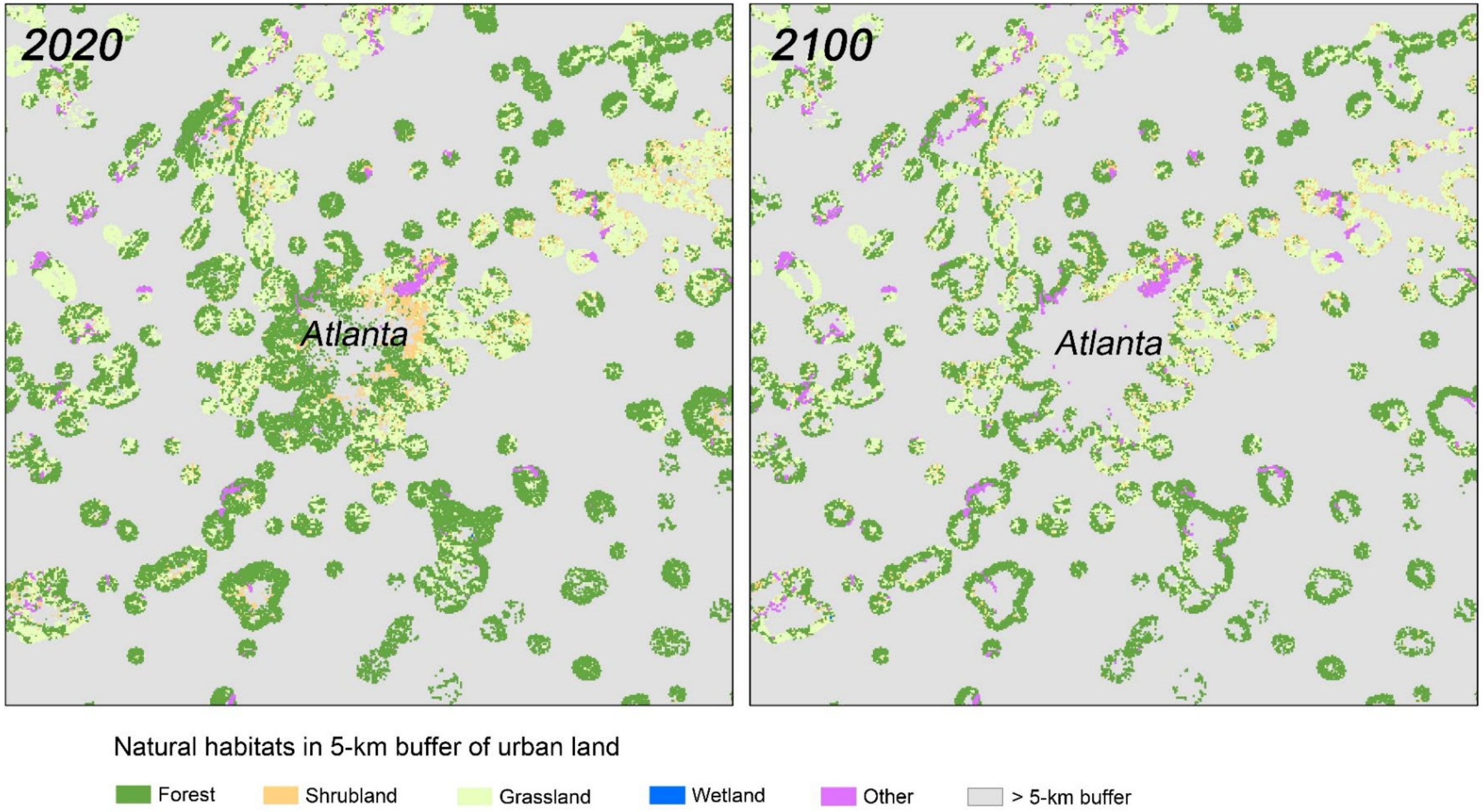

Supplementary Fig. 32 Illustration of habitat fragmentation changes due to future urban expansion. Case of Atlanta in the US between 2020 and 2100 under SSP5.

Supplementary Table 1. Reclassification of ESA-CCI land cover classes in this study for identification of natural habitat.

ID	ESA-CCI and cover class	Cropland	Urban land	Natural habitat				
				Forest	Shrubland	Grassland	Wetland	Other
0	No data	0	0	0	0	0	0	1
10	Cropland, rainfed	1	0	0	0	0	0	0
20	Cropland, irrigated or post-flooding	1	0	0	0		0	0
30	Mosaic cropland (>50%) / natural vegetation (tree, shrub, herbaceous cover) (<50%)	1	0	0	0	0	0	0
40	Mosaic natural vegetation (tree, shrub, herbaceous cover) (>50%) / cropland (<50%)	1	0	0	0	0	0	0
50	Tree cover, broadleaved, evergreen, closed to open (>15%)	0	0	1	0	0	0	0
60	Tree cover, broadleaved, deciduous, closed to open (>15%)	0	0	1	0	0	0	0
70	Tree cover, needleleaved, evergreen, closed to open (>15%)	0	0	1	0	0	0	0
80	Tree cover, needleleaved, deciduous, closed to open (>15%)	0	0	1	0		0	0
90	Tree cover, mixed leaf type (broadleaved and needleleaved)	0	0	1	0	0	0	0
100	Mosaic tree and shrub (>50%) / herbaceous cover (<50%)	0	0	0	1	0	0	0
110	Mosaic herbaceous cover (>50%) / tree and shrub (<50%)	0	0	0	1	0	0	0
120	Shrubland	0	0	0	1	0	0	0
130	Grassland	0	0	0	0	1	0	0
140	Lichens and mosses	0	0	0	0	1	0	0
150	Sparse vegetation (tree, shrub, herbaceous cover) (<15%)	0	0	0	1	0	0	0
160	Tree cover, flooded, fresh or brakish water	0	0	1	0	0	0	0
170	Tree cover, flooded, saline water	0	0	1	0	0	0	0
180	Shrub or herbaceous cover, flooded, fresh/saline/brakish water	0	0	0	0	0	1	0
190	Urban areas	0	1	0	0	0	0	0
200	Bare areas	0	0	0	0	0	0	1
210	Water bodies	0	0	0	0	0	0	1
220	Permanent snow and ice	0	0	0	0	0	0	1

Note: This reclassification system was based on the Land Cover CCI product user guide version 2.0, van Vliet, J. (2019), and EUNIS Habitat Classification Revised 2004.

Supplementary Table 2. Estimated urban expansion-caused habitat loss areas across top 30 countries or regions by 2100.

Country or region name	ISO3	SSP1 (km^2)	SSP2 (km^2)	SSP3 (km^2)	SSP4 (km^2)	SSP5 (km^2)	Average of five SSPs (km^2)	Rank
United States	USA	7379006	6803376	1255513	3864061	17203878	7301167	1
Nigeria	NGA	603657	849182	1016400	1447065	754445	934150	2
Australia	AUS	808930	806035	216312	586775	1689172	821445	3
Germany	DEU	473524	404182	24306	199999	1294032	479209	4
United Kingdom	GBR	483359	441249	38969	239744	1089061	458476	5
Saudi Arabia	SAU	264804	363380	454780	449952	409465	388476	6
Canada	CAN	351989	337725	54869	205145	971988	384343	7
Iran	IRN	228015	319773	475741	516259	255778	359113	8
Brazil	BRA	245463	353711	697405	194870	267456	351781	9
China	CHN	359564	300081	229957	299938	406636	319235	10
India	IND	241471	285014	340881	284342	284159	287174	11
Mexico	MEX	165534	274911	650901	150053	145618	277403	12
South Africa	ZAF	268738	278488	320087	142797	359461	273914	13
New Zealand	NZL	198262	193526	31322	127575	556712	221479	14
United Arab Emirates	ARE	125393	201459	261952	262137	228210	215830	15

France	FRA	215573	190103	12033	90405	560738	213770	16
Norway	NOR	187078	176545	32344	107201	517276	204089	17
Chad	TCD	53273	136078	198399	386373	135670	181958	18
Sudan	SDN	158103	170603	180203	219477	156003	176878	19
Spain	ESP	171709	155894	9322	80611	406357	164779	20
Netherlands	NLD	154885	145219	8800	71991	397455	155670	21
Turkey	TUR	116856	166419	222324	106328	146663	151718	22
Switzerland	CHE	150225	135511	31667	98888	306211	144500	23
Italy	ITA	147471	129791	8517	65851	363475	143021	24
Guatemala	GTM	78957	112957	196014	230657	64457	136608	25
Venezuela	VEN	97942	139302	254193	91771	97520	136146	26
Iraq	IRQ	79078	114177	178162	194832	94673	132184	27
Russia	RUS	108168	199634	29692	71331	247948	131355	28
Zambia	ZMB	94951	105160	109695	133288	94483	107515	29
Zimbabwe	ZWE	90061	100061	104507	138291	87673	104119	30

Supplementary Table 3. Estimated urban expansion-caused habitat loss across different biome types around the world.

Biome name	Urban expansion-caused habitat loss and proportion of the biome area between 2015-2100 (km^2) under SSP scenarios				
	SSP1	SSP2	SSP3	SSP4	SSP5
Tropical and subtropical moist broadleaf forests	12705 (0.06)	15557 (0.08)	21011 (0.11)	18226 (0.09)	15921 (0.08)
Tropical and subtropical dry broadleaf forests	1300 (0.04)	1783 (0.06)	3165 (0.11)	1550 (0.05)	1767 (0.06)
Tropical and subtropical coniferous forests	967 (0.14)	1420 (0.20)	2671 (0.38)	1860 (0.26)	847 (0.12)
Temperate broadleaf and mixed forests	74174 (0.58)	68929 (0.54)	15169 (0.12)	40635 (0.32)	174038 (1.36)
Temperate coniferous forests	12589 (0.31)	11394 (0.28)	2120 (0.05)	6425 (0.16)	32805 (0.80)
Boreal forests/taiga	1962 (0.01)	2128 (0.01)	291 (0.00)	1004 (0.01)	5883 (0.04)
Tropical and subtropical grasslands, savannas, and shrublands	13515 (0.07)	15410 (0.08)	15754 (0.08)	19334 (0.1)	16975 (0.08)
Temperate grasslands, savannas, and shrublands	12633 (0.13)	12406 (0.12)	5945 (0.06)	8502 (0.08)	25435 (0.25)
Flooded grasslands and savannas	1158 (0.11)	1816 (0.17)	2285 (0.21)	3943 (0.36)	2017 (0.18)
Montane grasslands and shrublands	2640 (0.05)	2862 (0.06)	3411 (0.07)	2162 (0.04)	3144 (0.06)
Tundra	226 (0.00)	215 (0.00)	29 (0.00)	134 (0.00)	668 (0.01)
Mediterranean forests, woodlands, and scrub	10403 (0.32)	10825 (0.34)	6739 (0.21)	7962 (0.25)	19881 (0.62)
Deserts and xeric shrublands	16558 (0.06)	21102 (0.08)	26237 (0.09)	22771 (0.08)	24958 (0.09)
Mangroves	1136 (0.33)	1336 (0.39)	1894 (0.55)	1417 (0.41)	1254 (0.36)

Supplementary Table 4. Spatial overlap between future urban expansion and conservation prioritization schemes.

SSP scenarios	Year	Protected areas (km^2)	Biodiversity hotspots (km^2)	Global 200 (km^2)	Last of the Wild (km^2)
	2015	30594 (0.0564)	228321 (0.9161)	210807 (0.3838)	1684 (0.0030)
**SSP1**	2020	30944 (0.0571)	246107 (0.9875)	228995 (0.4169)	1689 (0.0030)
	2030	35753 (0.0659)	275089 (1.1038)	258234 (0.4701)	1701 (0.0030)
	2040	40444 (0.0746)	299801(1.2029)	282010 (0.5134)	1708 (0.0030)
	2050	44584 (0.0822)	318726 (1.2789)	299302 (0.5449)	1722 (0.0030)
	2060	48165 (0.0888)	333257 (1.3372)	313066 (0.5699)	1733 (0.0031)
	2070	50873 (0.0938)	344443 (1.3821)	323420 (0.5888)	1736 (0.0031)
	2080	52881 (0.0975)	352846 (1.4158)	331296 (0.6031)	1752 (0.0031)
	2090	54250 (0.1001)	358256 (1.4375)	336018 (0.6117)	1755 (0.0031)
	2100	54786 (0.1010)	360408 (1.4461)	337992 (0.6153)	1757 (0.0031)
**SPP2**	2020	30674 (0.0566)	244722 (0.9819)	227623 (0.4144)	1687 (0.0030)
	2030	34931 (0.0644)	272655 (1.0940)	255717 (0.4655)	1700 (0.0030)
	2040	39179 (0.0723)	297369 (1.1932)	279259 (0.5084)	1706 (0.0030)
	2050	42899 (0.0791)	317405 (1.2736)	297320 (0.5413)	1724 (0.0030)
	2060	46401 (0.0856)	334738 (1.3431)	312644 (0.5692)	1751 (0.0031)
	2070	49458 (0.0912)	349205 (1.4012)	325499 (0.5926)	1760 (0.0031)
	2080	52234 (0.0963)	360202 (1.4453)	335440 (0.6107)	1778 (0.0031)
	2090	54414 (0.1004)	369078 (1.4809)	343001 (0.6244)	1789 (0.0032)

	2100	56056 (0.1034)	375203 (1.5055)	348131 (0.6338)	1789 (0.0032)
**SPP3**	2020	30069 (0.0555)	242064 (0.9713)	224739 (0.4091)	1687 (0.0030)
	2030	32237 (0.0595)	264674 (1.0620)	246405 (0.4486)	1700 (0.0030)
	2040	34171 (0.0630)	283442 (1.1373)	262872 (0.4785)	1706 (0.0030)
	2050	36029 (0.0664)	300366 (1.2052)	276513 (0.5034)	1727 (0.0030)
	2060	38054 (0.0702)	315329 (1.2652)	288230 (0.5247)	1758 (0.0031)
	2070	40044 (0.0739)	330006 (1.3241)	299013 (0.5443)	1767 (0.0031)
	2080	42192 (0.0778)	344017 (1.3804)	310013 (0.5644)	1781 (0.0031)
	2090	44334 (0.0818)	357510 (1.4345)	321023 (0.5844)	1784 (0.0031)
	2100	46705 (0.0861)	372233 (1.4936)	332959 (0.6061)	1843 (0.0033)
**SPP4**	2020	30745 (0.0567)	245799 (0.9863)	228681 (0.4163)	1694 (0.0030)
	2030	34737 (0.0641)	273236 (1.0963)	255875 (0.4658)	1705 (0.0030)
	2040	38462 (0.0709)	295756 (1.1867)	276066 (0.5026)	1725 (0.0030)
	2050	42206 (0.0778)	314099 (1.2603)	290932 (0.5296)	1751 (0.0031)
	2060	45577 (0.0841)	329310 (1.3213)	302878 (0.5514)	1768 (0.0031)
	2070	48748 (0.0899)	343022 (1.3764)	313220 (0.5702)	1816 (0.0032)
	2080	50068 (0.0923)	355143 (1.4250)	321465 (0.5852)	1865 (0.0033)
	2090	50726 (0.0936)	365389 (1.4661)	328148 (0.5974)	1871 (0.0033)
	2100	51347 (0.0947)	375428 (1.5064)	334912 (0.6097)	1897 (0.0033)
**SPP5**	2020	31195 (0.0575)	247218 (0.9920)	229987 (0.4187)	1688 (0.0030)

2030	37079 (0.0684)	280665 (1.1262)	263861 (0.4803)	1702 (0.0030)
2040	43689 (0.0806)	311238 (1.2488)	293873 (0.5350)	1712 (0.0030)
2050	50950 (0.0940)	339310 (1.3615)	321001 (0.5844)	1730 (0.0031)
2060	58604 (0.1081)	365214 (1.4654)	346313 (0.6304)	1747 (0.0031)
2070	66326 (0.1223)	389573 (1.5631)	370184 (0.6739)	1761 (0.0031)
2080	74238 (0.1369)	412328 (1.6544)	393178 (0.7158)	1772 (0.0031)
2090	82181 (0.1516)	433147 (1.7380)	414532 (0.7546)	1781 (0.0031)
2100	89901 (0.1658)	452478 (1.8156)	434115 (0.7903)	1785 (0.0031)

Note: Percentage overlap of future urban expansion with conservation prioritization schemes are presented in parentheses.

Supplementary Table 5. Estimated urban expansion-caused edge distance change areas across top 30 countries or regions by 2100.

Country or region name	ISO3	SSP1(km^2)	SSP2(km^2)	SSP3(km^2)	SSP4(km^2)	SSP5(km^2)	Average of five SSPs (km^2)	Rank
Mauritania	MRT	121065	77786	78330	77484	77387	86410	1
Algeria	DZA	57204	52514	56468	52854	53020	54412	2
Saudi Arabia	SAU	31939	38142	73791	72196	40529	51319	3
Western Sahara	ESH	57226	40494	46822	39717	39978	44847	4
United States	USA	27497	25473	3848	14251	64922	27198	5
Yemen	YEM	5805	5919	18121	14342	7574	10352	6
Iran	IRN	6528	9718	13366	13723	7693	10206	7
Egypt	EGY	8642	8183	11134	7272	7653	8577	8
Mexico	MEX	3284	4975	11904	2271	2183	4923	9
Nigeria	NGA	3522	4597	4161	6768	3580	4526	10
Australia	AUS	4272	4354	1290	3029	7782	4145	11
Iraq	IRQ	2094	3407	5424	5873	2353	3830	12
New Zealand	NZL	3924	3655	889	2628	6741	3567	13
United Arab Emirates	ARE	1917	3008	3656	4157	3016	3151	14
Kuwait	KWT	2279	2841	3193	3204	3107	2925	15

Jordan	JOR	1783	2466	3860	3807	1917	2767	16
Kenya	KEN	90	3846	3880	3897	1133	2569	17
Caspian Sea	XCA	2003	1605	4096	2032	2105	2368	18
South Africa	ZAF	2031	2205	2532	1056	2733	2111	19
Argentina	ARG	1179	2081	4573	1050	1206	2018	20
Oman	OMN	1419	2075	2175	2305	2101	2015	21
United Kingdom	GBR	2276	1973	127	1054	4089	1904	22
Canada	CAN	1366	1372	190	781	5207	1783	23
Libya	LBY	1413	574	3199	3285	434	1781	24
Kazakhstan	KAZ	1536	1561	2290	1526	1548	1692	25
Sudan	SDN	1196	1631	1679	2075	1634	1643	26
Chile	CHL	1321	1651	2762	967	1119	1564	27
Brazil	BRA	912	1585	3448	617	1027	1518	28
Ecuador	ECU	1043	1110	2614	1135	1116	1404	29
Cameroon	CMR	816	971	1376	2405	1380	1390	30

Supplementary Table 6. Conservation priority ecoregions for future urban-caused habitat and species loss.

Ecoregions Name	Urban area in 2015 (km^2)	Projected mean urban growth area by 2100 (km^2, SSP1-SSP5)	Projected mean urban growth rate by 2100 (SSP1-SSP5)	Mean small-ranged species number of vertebrates
Albertine Rift montane forests	219	59.80	27	63
Cameroonian Highlands forests	133	142.80	107	42
Cauca Valley montane forests	145	149.20	103	127
Central American dry forests	635	213.80	34	40
Central American montane forests	142	175.00	123	76
Central American pine-oak forests	993	1101.00	111	61
Central Andean wet puna	431	134.20	31	61
Cordillera La Costa montane forests	252	113.60	45	71
Costa Rican seasonal moist forests	384	171.60	45	56
East African montane forests	51	145.20	285	43
Eastern Cordillera real montane forests	344	77.20	22	153
Hispaniolan moist forests	622	166.40	27	43
Isthmian-Atlantic moist forests	223	60.80	27	94
La Costa xeric shrublands	839	480.80	57	34
Madagascar subhumid forests	186	363.00	195	41
Magdalena Valley dry forests	61	51.20	84	62
Magdalena Valley montane forests	920	407.60	44	131
Northwestern Andean montane forests	519	146.20	28	170
Paraguana xeric scrub	223	144.00	65	45
Peruvian Yungas	221	49.80	23	123

Petén-Veracruz moist forests	397	190.80	48	52
Puerto Rican dry forests	138	131.80	96	38
Puerto Rican moist forests	978	853.00	87	45
Serra do Mar coastal forests	5226	1097.46	21	53
Sierra Madre de Chiapas moist forests	77	122.40	159	65
Southern Atlantic mangroves	746	222.60	30	38
Southern Pacific dry forests	437	129.60	30	49
Trans-Mexican Volcanic Belt pine-oak forests	1145	250.60	22	45
Tumbes-Piura dry forests	110	65.20	59	73
Venezuelan Andes montane forests	188	59.20	31	120

Supplementary Table 7. Estimated urban expansion-caused average species richness loss per 10-km grid cell across top 30 countries or regions by 2100.

Country or region name	ISO3	SSP1	SSP2	SSP3	SSP4	SSP5	Average of five SSPs	Rank
Kenya	KEN	40	39	39	42	38	39	1
Swaziland	SWZ	28	36	38	36	32	34	2
Brunei	BRN	34	34	35	24	37	33	3
Zambia	ZMB	30	32	33	36	30	32	4
Republic of Congo	COG	28	28	28	34	30	30	5
Zimbabwe	ZWE	28	29	29	34	27	29	6
Malawi	MWI	26	27	27	33	25	28	7
Gambia	GMB	22	26	26	41	23	28	8
Chad	TCD	19	27	30	32	28	27	9
Nigeria	NGA	23	25	27	34	24	27	10
Mozambique	MOZ	23	25	27	31	23	26	11
Cameroon	CMR	25	24	25	30	24	26	12
Liberia	LBR	16	21	26	42	17	24	13
Togo	TGO	18	22	27	34	20	24	14

Uganda	UGA	22	22	23	27	22	23	15
Tanzania	TZA	21	22	23	25	21	22	16
Gabon	GAB	20	21	21	22	20	21	17
Senegal	SEN	17	20	20	28	18	20	18
Benin	BEN	17	19	20	26	18	20	19
Rwanda	RWA	18	18	20	24	19	20	20
Ghana	GHA	17	18	19	26	16	19	21
Equatorial Guinea	GNQ	18	18	21	20	16	19	22
Sierra Leone	SLE	16	16	17	26	16	18	23
Guinea	GIN	14	15	16	28	13	17	24
Liechtenstein	LIE	19	17	3	12	32	17	25
Trinidad and Tobago	TTO	11	16	33	11	12	17	26
Burundi	BDI	16	14	16	22	14	16	27
Vatican City	VAT	19	18	6	16	20	16	28
Côte d'Ivoire	CIV	15	15	16	19	14	16	29
Suriname	SUR	11	13	34	10	8	15	30

Supplementary Table 8. Summary of assumptions about urbanization patterns for five SSPs.

Elements	SSP1	SSP2	SPP3	SSP4	SSP5
Urbanization in high- income countries	Fast	Central	Slow	Central	Fast
Urbanization in medium-income countries	Fast	Central	Slow	Fast	Fast
Urbanization in low-income countries	Fast	Central	Slow	Fast	Fast
Urbanization rate by 2100	92.6%	79.7%	58.4%	91.7%	93.0%
Spatial pattern	Concentrated	Historical patterns	Mixed	Mixed	Sprawl
Migration	Moderate	Intermediate	Low	Mixed	Fast

Supplementary Table 9. Assumptions for urban planning and protected areas under SSPs.

Scenarios	Urban planning assumptions	Protected areas assumptions (Environment assumptions)
SSP1	Urbanization is well managed, and urban planning is promoted in tandem with high urbanization rates.	It is an environment-friendly development pattern to strengthen the protection of fragile ecosystems and regions such as protected areas. Land use is strictly regulated. Urban expansion has barely encroached on protected areas. Protected areas are effective. *Protected areas are on track to meet Aichi's 17% target due to strong land-use change regulation.*
SSP2	Moderate urban planning regulation.	Growing energy demand has led to the continuous environmental deterioration. Moderate regulation of land use leads to a slow decline in deforestation rates. Moderate land use regulation makes the effectiveness of protected areas in the middle level. *Protected areas are being moderately encroached upon. Protected areas are expected to meet the Aichi target of 17% of land area due to moderate land use change regulation gradually implemented from 2010–2050.*
SSP3	Urban settlements are poorly planned, particularly in developing countries where inequality and fragmentation cause mixed pattern of urban change.	Not enough attention has been paid to solving environmental problems, resulting in serious environmental degradation in some areas. Deforestation continues because of a lack of regulation, competition for land and the rapid expansion of agriculture. Poor land use regulation leads to low effectiveness of protected areas. Urban expansion has encroached heavily on protected areas. *Protected areas are under serious threat.*

SSP4	Spatial development pattern varies across cities, with urban sprawl dominating in some cities, whereas better planning in cities that are predominantly inhabited by the higher-income groups leads to more concentrated development.	There are significant differences in environmental conditions. On the one hand, there are some areas of world concern, close to the places where middle- and high-income groups live and vacation, which are well managed. On the other hand, resource and production areas and many other out of sight places are neglected and become deteriorated. *Conservation of protected areas is also divided, with highly regulated and well-managed areas in middle- and high-income countries, but largely unmanaged and deteriorating areas in low-income countries.*
SSP5	It is difficult for urban planning to keep up with high urbanization rates, and the sprawling pattern of development is dominant.	Regulations are imperfect, and many protected areas are not effectively protected. *Protected areas are under serious threat.*

Supplementary Table 10. Modelled mean estimates (following Newbold et al. (2015), they based on PREDICTS) of relative percent biodiversity change for each Land System.

code	Land System	Species Richness (SR)	SR-min	SR-max	Abundance (CI)	CI-min	CI-max	Change-SR (%)	Change-SR-min (%)	Change-SR-max (%)	Change-CI (%)	Change-CI-min (%)	Change-CI-max (%)
0	Cropland ext, few ls	71.85	62.65	82.35	80.80	62.60	104.20	31	20	40	53	36	66
1	Cropland ext, bgs	68.00	57.40	80.70	77.25	56.65	105.70	27	18	35	51	36	63
2	Cropland ext, pp	68.00	57.40	80.70	77.25	56.65	105.70	27	18	35	51	36	63
3	Cropland med. Int, few ls	66.25	56.85	77.20	63.55	48.05	84.05	25	15	34	41	20	56
4	Cropland med. int, bgs	62.40	51.60	75.55	60.00	42.10	85.55	20	13	27	37	21	50
5	Cropland med. int, pp	62.40	51.60	75.55	60.00	42.10	85.55	20	13	27	37	21	50
6	Cropland int, few ls	67.15	56.95	79.25	70.45	51.55	96.60	26	17	34	47	30	59
7	Cropland int, bgs	63.30	51.70	77.60	66.90	45.60	98.10	21	15	27	44	31	54
8	Cropland int, pp	63.30	51.70	77.60	66.90	45.60	98.10	21	15	27	44	31	54
14	Mosaic cropland and forest, pp	63.30	51.70	77.60	66.90	45.60	98.10	21	15	27	44	31	54
15	Mosaic cropland ext and open forest, few ls	71.85	62.65	82.35	80.80	62.60	104.20	31	20	40	53	36	66
16	Mosaic cropland m. int and open forest, few ls	66.25	56.85	77.20	63.55	48.05	84.05	25	15	34	41	20	56
17	Mosaic cropland int and open forest, few ls	67.15	56.95	79.25	70.45	51.55	96.60	26	17	34	47	30	59
18	Dense forest	96.13	90.54	100.39	98.03	88.28	108.28	48	34	59	62	38	76
19	Open forest, few ls	89.23	79.54	100.19	90.96	71.44	104.63	44	34	53	59	36	70
20	Open forest, pp	88.00	75.34	102.92	97.56	70.81	122.80	43	36	50	61	45	70
21	Mosaic grassland and open forest	96.13	90.54	100.39	98.03	88.28	108.28	48	34	59	62	38	76
22	Mosaic grassland and bare	96.13	90.54	100.39	98.03	88.28	108.28	48	34	59	62	38	76
23	Natural grassland	96.13	90.54	100.39	98.03	88.28	108.28	48	34	59	62	38	76
24	Grassland, few ls	70.60	61.30	81.20	72.20	56.00	93.00	29	19	39	48	28	62

25	Grassland, bgs	62.90	50.80	77.90	65.10	44.10	96.00	21	15	26	42	30	52
27	Bare,few ls	70.60	61.30	81.20	72.20	56.00	93.00	29	19	39	48	28	62
28	Peri-urban & villages	96.00	79.40	116.00	81.80	51.60	129.70	48	43	53	54	48	59
29	Urban	49.80	37.50	66.00	37.60	21.10	67.20	0	0	0	0	0	0
30	Dense forest_CS	96.13	90.54	100.39	98.03	88.28	108.28	48	34	59	62	38	76
31	Open forest, few ls_CS	89.23	79.54	100.19	90.96	71.44	104.63	44	34	53	59	36	70
32	Open forest, pp_CS	88.00	75.34	102.92	97.56	70.81	122.80	43	36	50	61	45	70
33	Mosaic grassland and open forest_CS	96.13	90.54	100.39	98.03	88.28	108.28	48	34	59	62	38	76
34	Mosaic grassland and bare_CS	96.13	90.54	100.39	98.03	88.28	108.28	48	34	59	62	38	76
35	Natural grassland_CS	96.13	90.54	100.39	98.03	88.28	108.28	48	34	59	62	38	76
36	Grassland, few ls_CS	70.60	61.30	81.20	72.20	56.00	93.00	29	19	39	48	28	62
37	Grassland, bgs_CS	62.90	50.80	77.90	65.10	44.10	96.00	21	15	26	42	30	52

Note: The values represent the percentage of remaining biodiversity expressed in terms of local species richness (SR) and abundance (CI). All values are relative to an unaffected baseline (primary vegetation, minimum intensity of use, zero population density, and maximum observed distance to a road and travel time to major city). The first number gives the mean estimate of the modeling, and the minimum and maximum numbers give a 95% confidence limit (excluding about 10% of the study data at a time under ten-fold cross-validation). ext = extensive, med. Int = medium intensity, int = intensity, ls = livestock, bgs = cattle, goat and sheep, pp = pig and poultry. Where Change-SR and Change-CI are the percent change in species richness and species richness from the original land system type to the urban land use type, respectively. Change-min and change-max also are 95% confidence limits.

Supplementary References:

1. Jiang, L. & O'Neill, B. C. Global urbanization projections for the Shared Socioeconomic Pathways. *Global Environmental Change* **42**, 193-199, doi:10.1016/j.gloenvcha.2015.03.008 (2017).
2. Gossop, C. Low carbon cities: An introduction to the special issue. *Cities* **28**, 495-497, doi:10.1016/j.cities.2011.09.003 (2011).
3. Ewing, R. & Cervero, R. Travel and the Built Environment. *Journal of the American Planning Association* **76**, 265-294, doi:10.1080/01944361003766766 (2010).
4. Jones, B. & O'Neill, B. C. Spatially explicit global population scenarios consistent with the Shared Socioeconomic Pathways. *Environmental Research Letters* **11**, 084003, doi:10.1088/1748-9326/11/8/084003 (2016).
5. O'Neill, B. C. *et al.* The roads ahead: Narratives for shared socioeconomic pathways describing world futures in the 21st century. *Global Environmental Change* **42**, 169-180, doi:10.1016/j.gloenvcha.2015.01.004 (2017).
6. Mohan, R. The effect of population growth, the pattern of demand and of technology on the process of urbanization. *Journal of Urban Economics* **15**, 125-156, doi:10.1016/0094-1190(84)90011-1 (1984).
7. Kelley, A. C. & Williamson, J. G. Population Growth, Industrial Revolutions, and the Urban Transition. *Population and Development Review* **10**, 419-441, doi:10.2307/1973513 (1984).
8. Ledent, J. Rural-Urban Migration, Urbanization, and Economic Development. *Economic Development and Cultural Change* **30**, 507-538, doi:10.1086/452574 (1982).
9. Abdel-Rahman, A. N., Safarzadeh, M. R. & Bottomley, M. B. Economic growth and urbanization: A cross-section and time-series analysis of thirty-five developing countries. *International Review of Economics and Business* **53**, 334-348 (2006).
10. Jiang, L. Internal consistency of demographic assumptions in the shared socioeconomic pathways. *Population and Environment* **35**, 261-285, doi:10.1007/s11111-014-0206-3 (2014).
11. Fay, M. & Opal, C. *Urbanization without growth: A not so uncommon phenomenon*. Vol. 2412 (World Bank Publications, 2000).
12. Chen, G. *et al.* Global projections of future urban land expansion under shared socioeconomic pathways. *Nature Communications* **11**, doi:10.1038/s41467-020-14386-x (2020).
13. Kriegler, E. *et al.* Fossil-fueled development (SSP5): An energy and resource intensive scenario for the 21st century. *Global Environmental Change* **42**, 297-315 (2017).
14. Fricko, O. *et al.* The marker quantification of the Shared Socioeconomic Pathway 2: A middle-of-the-road scenario for the 21st century. *Global Environmental*

Change **42**, 251-267 (2017).
15. van Vuuren, D. P. *et al.* Energy, land-use and greenhouse gas emissions trajectories under a green growth paradigm. *Global Environmental Change* **42**, 237-250 (2017).
16. Calvin, K. *et al.* The SSP4: A world of deepening inequality. *Global Environmental Change* **42**, 284-296 (2017).
17. Fujimori, S. *et al.* SSP3: AIM implementation of Shared Socioeconomic Pathways. *Global Environmental Change* **42**, 268-283 (2017).
18. UNEP-WCMC & IUCN. *The World Database on Protected Areas (WDPA). https:// www.protectedplanet.net/.* Accessed 15 May 2020).
19. Jones, K. R. *et al.* One-third of global protected land is under intense human pressure. *Science* **360**, 788-791, doi:10.1126/science.aap9565 (2018).
20. Huang, X. *et al.* 30 m global impervious surface area dynamics and urban expansion pattern observed by Landsat satellites: From 1972 to 2019. *Science China Earth Sciences* **64**, 1922-1933, doi:10.1007/s11430-020-9797-9 (2021).
21. Liu, X. *et al.* High-spatiotemporal-resolution mapping of global urban change from 1985 to 2015. *Nature Sustainability*, doi:10.1038/s41893-020-0521-x (2020).
22. Gong, P. *et al.* Annual maps of global artificial impervious area (GAIA) between 1985 and 2018. *Remote Sensing of Environment* **236**, 111510, doi:10.1016/j.rse.2019.111510 (2020).
23. Pesaresi, M. *et al.* Operating procedure for the production of the Global Human Settlement Layer from Landsat data of the epochs 1975, 1990, 2000, and 2014. *Publications Office of the European Union*, 1-62 (2016).
24. Myers, N., Mittermeier, R. A., Mittermeier, C. G., da Fonseca, G. A. B. & Kent, J. Biodiversity hotspots for conservation priorities. *Nature* **403**, 853-858, doi:10.1038/35002501 (2000).
25. Hoffman, M., Koenig, K., Bunting, G., Costanza, J. & Williams, K. J. Biodiversity Hotspots (version 2016.1). *Zenodo.* https://doi.org/10.5281/zenodo.3261807 (2016).
26. Olson, D. M. & Dinerstein, E. The Global 200: Priority Ecoregions for Global Conservation. *Annals of the Missouri Botanical Garden* **89**, 199-224, doi:10.2307/3298564 (2002).
27. Wildlife Conservation Society - WCS & Center for International Earth Science Information Network - CIESIN - Columbia University. (NASA Socioeconomic Data and Applications Center (SEDAC), Palisades, NY, 2005).
28. Hudson, L. N. *et al.* The PREDICTS database: a global database of how local terrestrial biodiversity responds to human impacts. *Ecology and Evolution* **4**, 4701-4735, doi:10.1002/ece3.1303 (2014).
