# Supplementary material for: Global impacts of future urban expansion on terrestrial vertebrate diversity
Source: Nat Commun. 2022 Mar 25;13:1628. doi: 10.1038/s41467-022-29324-2 (PMC8956596; doi:10.1038/s41467-022-29324-2)
Supplement: Supplementary file 3 — Reporting Summary [file 41467_2022_29324_MOESM3_ESM.pdf]

nature portfolio

Corresponding author(s): Guangdong Li

Last updated by author(s): Feb 5, 2022

# Reporting Summary

Nature Portfolio wishes to improve the reproducibility of the work that we publish. This form provides structure for consistency and transparency in reporting. For further information on Nature Portfolio policies, see our Editorial Policies and the Editorial Policy Checklist.

## Statistics

For all statistical analyses, confirm that the following items are present in the figure legend, table legend, main text, or Methods section.

| n/a | Confirmed | |
|---|---|---|
| ☐ | ☒ | The exact sample size ($n$) for each experimental group/condition, given as a discrete number and unit of measurement |
| ☒ | ☐ | A statement on whether measurements were taken from distinct samples or whether the same sample was measured repeatedly |
| ☐ | ☒ | The statistical test(s) used AND whether they are one- or two-sided<br>*Only common tests should be described solely by name; describe more complex techniques in the Methods section.* |
| ☐ | ☒ | A description of all covariates tested |
| ☐ | ☒ | A description of any assumptions or corrections, such as tests of normality and adjustment for multiple comparisons |
| ☐ | ☒ | A full description of the statistical parameters including central tendency (e.g. means) or other basic estimates (e.g. regression coefficient) AND variation (e.g. standard deviation) or associated estimates of uncertainty (e.g. confidence intervals) |
| ☐ | ☒ | For null hypothesis testing, the test statistic (e.g. $F$, $t$, $r$) with confidence intervals, effect sizes, degrees of freedom and $P$ value noted<br>*Give P values as exact values whenever suitable.* |
| ☒ | ☐ | For Bayesian analysis, information on the choice of priors and Markov chain Monte Carlo settings |
| ☐ | ☒ | For hierarchical and complex designs, identification of the appropriate level for tests and full reporting of outcomes |
| ☒ | ☐ | Estimates of effect sizes (e.g. Cohen's $d$, Pearson's $r$), indicating how they were calculated |

*Our web collection on statistics for biologists contains articles on many of the points above.*

## Software and code

Policy information about availability of computer code

| | |
|---|---|
| Data collection | All methods of data collection in this study are described in the paper. |
| Data analysis | All methods of data analysis in this study are described in the paper. We used the ArcGIS Pro v2.5 software and FRAGSTATS v4.2 software to analyze the data in this manuscript. |

For manuscripts utilizing custom algorithms or software that are central to the research but not yet described in published literature, software must be made available to editors and reviewers. We strongly encourage code deposition in a community repository (e.g. GitHub). See the Nature Portfolio guidelines for submitting code & software for further information.

## Data

Policy information about availability of data

All manuscripts must include a data availability statement. This statement should provide the following information, where applicable:

- Accession codes, unique identifiers, or web links for publicly available datasets
- A description of any restrictions on data availability
- For clinical datasets or third party data, please ensure that the statement adheres to our policy

All data in this study, including simulated data, are available following the descriptions in the paper. The future urban expansion data can be obtained from https://doi.org/10.1594/PANGAEA.905890. The ESA CCI Land Cover product can be found at http://maps.elie.ucl.ac.be/CCI/viewer/download.php. The SSP database is available from https://tntcat.iiasa.ac.at/SspDb. The species richness data can be obtained from https://biodiversitymapping.org/index.php/download/. The updated land-systems map is publicly downloadable at https://box.hu-berlin.de/d/053f45f377/?p=%2FKehoe_et-al_2017_NatureEcoEvo&mode=list. Data on the boundaries of protected areas are retrieved from the World Database on Protected Areas (May 2020, https://www.protectedplanet.net/en). The biodiversity hotspots data can be retrieved from https://zenodo.org/record/3261807#.YToUlJ0zYuU. The Global 200 data is available from https://www.worldwildlife.org/publications/global-200.

The Last of the Wild Areas data (version 2.0) is available from https://sedac.ciesin.columbia.edu/data/set/wildareas-v2-last-of-the-wild-geographic. The PREDICTS database is available from https://data.nhm.ac.uk/dataset/the-2016-release-of-the-predicts-database.

# Field-specific reporting

Please select the one below that is the best fit for your research. If you are not sure, read the appropriate sections before making your selection.

☐ Life sciences ☐ Behavioural & social sciences ☒ Ecological, evolutionary & environmental sciences

For a reference copy of the document with all sections, see nature.com/documents/nr-reporting-summary-flat.pdf

# Ecological, evolutionary & environmental sciences study design

All studies must disclose on these points even when the disclosure is negative.

| | |
|---|---|
| Study description | This study integrated comprehensive scenario (five shared socioeconomic pathways, SSPs) analyses of future urban expansion and datasets on habitat and terrestrial biodiversity (amphibians, mammals, and birds) to quantify the impact of future urban expansion on global terrestrial biodiversity. |
| Research sample | We obtained fine-scale range maps for terrestrial vertebrates using data on >22,000 species of amphibians, mammals, and birds (three well-known terrestrial vertebrate taxa). For each taxon, local richness for all species, small-ranged species, and threatened species listed by the International Union for the Conservation of Nature were identified. Complete global datasets including all countries and all land areas were used to analyze the impact of future spatially-explicit urban expansion on natural habitat and terrestrial biodiversity. To examine the effects of urban land changes on habitat fragmentation, we established a 5-km buffer around urban land in 2100 across the SSP scenarios and measured the changes in landscape metrics of natural habitat within this buffer. |
| Sampling strategy | Our sample was all terrestrial amphibians, mammals, and birds as assessed by the IUCN Red List and BirdLife International (2018 update). |
| Data collection | Data was obtained from the published datasets as described in the Data availability statement. Guangdong Li recorded the data. Data were downloaded from online public repositories. |
| Timing and spatial scale | 2020-2100 with an interval of 10 years; Global scale. |
| Data exclusions | No data were excluded from the analyses. |
| Reproducibility | The calculation of all results can be verified and reproduced by the data and softwares supplied as stated in the manuscript. |
| Randomization | We selected all data available, and random selection was not performed. |
| Blinding | Blinding was not relevant for our study because there were no deciding tasks done in this study. |

Did the study involve field work? ☐ Yes ☒ No

# Reporting for specific materials, systems and methods

We require information from authors about some types of materials, experimental systems and methods used in many studies. Here, indicate whether each material, system or method listed is relevant to your study. If you are not sure if a list item applies to your research, read the appropriate section before selecting a response.

## Materials & experimental systems

| n/a | Involved in the study | |
|---|---|---|
| ☒ | ☐ | Antibodies |
| ☒ | ☐ | Eukaryotic cell lines |
| ☒ | ☐ | Palaeontology and archaeology |
| ☒ | ☐ | Animals and other organisms |
| ☒ | ☐ | Human research participants |
| ☒ | ☐ | Clinical data |
| ☒ | ☐ | Dual use research of concern |

## Methods

| n/a | Involved in the study | |
|---|---|---|
| ☒ | ☐ | ChIP-seq |
| ☒ | ☐ | Flow cytometry |
| ☒ | ☐ | MRI-based neuroimaging |
